# Using MUC2 mucin producing tumorigenic human goblet-like cells to uncover functional properties of the mucus barrier

**DOI:** 10.1080/19490976.2025.2542385

**Published:** 2025-08-08

**Authors:** Hayley Gorman, France Moreau, Elise Beaupré, Nitin Nitin, Wesley F. Zandberg, Kirk Bergstrom, Paul M. K. Gordon, Antoine Dufour, Kris Chadee

**Affiliations:** a Departments of Microbiology, Immunology and Infectious Diseases, University of Calgary, Calgary, Alberta, Canada; b Department of Chemistry, University of British Columbia Okanagan, Kelowna, British Columbia, Canada; c Department of Biology, University of British Columbia Okanagan, Kelowna, British Columbia, Canada; d Centre for Health Genomics and Informatics, Snyder Institute for Chronic Diseases, University of Calgary, Calgary, Alberta, Canada; e Physiology and Pharmacology, University of Calgary, Calgary, Alberta, Canada; f Biochemistry and Molecular Biology, University of Calgary, Calgary, Alberta, Canada

**Keywords:** GI disease, host-pathogen interactions, MUC2 mucin, mucus hypersecretion

## Abstract

Goblet cells are the guardians that produce MUC2 mucin that forms the protective mucus barrier as the first line of innate host defense against pathogen invasion while sustaining a healthy gut microbiota. Using an unbiased screen, we cloned a high LS174T colonic adenocarcinoma MUC2 mucin-producing goblet-like cell that revealed by whole genome sequencing, mutations that upregulated the expression of tumor suppressor *TP53*, anterior gradient 2 (*AGR2*) and mitogen-activated protein kinase kinases (*MEK* and *MAPK*) genes that enhanced MUC2 biosynthesis and secretion. Hypersecretory *mutant* (*Mut*) cells exhibited a phenotype characterized by high constitutive *MUC2* mRNA, upregulation of glycosyltransferases, and hypersecretion of sialylated and fucosylated MUC2 mucus enumerated by glycomics analysis. *Mut* secreted mucus was altered quantified by increased permeability to fluorescence microsphere (0.2 μm and 1 μm) beads and to *Salmonella enterica* adherence, invasion, and cell death as compared to WT controls. Shotgun proteomic analysis revealed that *Mut* cells were upregulated in metabolic pathways for oxidative stress, HIF pathways and growth factors that enhanced wound healing, and tumorigenesis. These results highlight the importance of regulatory genes for proper mucus production in providing barrier function and homeostasis in the gastrointestinal tract that is markedly affected by metabolic stress and glycosylation prevalent in colonic carcinomas.

## Introduction

The colonic mucus bilayer is a critical component of the gastrointestinal tract. It acts as the first line of innate host defense against pathogen invasion by forming a physical barrier between the lumen and the underlying single layer of mucosal epithelial cells. Colonic mucus provides a nutrient rich substrate environment for host microbiota to colonize and, in turn, provide valuable nutrient release to the host.^1–3^ Each layer of the characteristic bilayer that forms in the colon has distinct functions.^4^ The outer mucus layer is loose and in direct contact with the host microbiota, providing its habitat and food source. Directly beneath the outer layer is a dense inner mucus layer that is impenetrable to microbiota under normal conditions and provides a nearly sterile layer between the microbiota-rich outer mucus layer and the colonic epithelium.^5^ When this mucus layer is disrupted or altered, it can lead to colonic infection, metabolic disorders, or inflammatory bowel disease (IBD).^6–8^

The major protein that comprises the mucus layer is a gel-forming protein, MUC2 mucin. This large molecular weight glycoprotein is specific to goblet cells, a subset of epithelial cells that specialize in forming the mucus layer. Within goblet cells, MUC2 mucin is packaged into specialized vesicles as mucin granules where it is stored, subsequently secreted into the lumen, and unfolds to form the protective mucus layer. The characteristic gel-like nature of mucus is due to the high levels of glycosylation in MUC2 mucin; the glycans on MUC2 bind water in the lumen and become sticky.^9–11^ High glycosylation of MUC2 is in part, what makes mucus a suitable substrate for microbiota; the high carbohydrate content provides a rich nutrient source.^12^ Specifically, the MUC2 mucin is highly *O*-linked glycosylated (up to 80% by weight) at its central core via proline, threonine, and serine residues (PTS),^3^ and lightly covered *N*-linked glycans at the *C*- and *N*-termini. The *O*-linked glycans in MUC2 include galactose (Gal), *N*-acetylgalactosamine (GalNAc), *N*-acetylglucosamine (GlcNAC), 5-N-acetylneuraminic acid (also known as sialic acid, Sia), and fucose (Fuc). *N*-glycans regulate MUC2 folding.^13^ The quantities of these various glycans, particularly *O*-glycans, play a critical role in the proper functioning of the protective mucus barrier as the extensive glycosylation provides protection from bacterial invasion in the gastrointestinal tract. Although bacteria have glycosidases that can degrade mucin glycans, this process occurs by removing single residues at a time. Therefore, the long and intricate glycosylation takes time to completely break down, allowing the mucus layer to be replenished simultaneously. Glycosylation on MUC2 occurs at the PTS domains and different, unique glycans extend onto the PTS domain to give a unique O-linked glycan repertoire.^14^ This extensive glycosylation creates a unique and varied glycan profile that allows the MUC2 mucus layer to be heterogeneous and cater to various microbiota,^14^ with over 100 unique glycan structures identified to date by mass spectrometry.^15,16^ Alterations in MUC2 quantities and glycan composition predispose the host to diseases such as IBD, cancer, or bacterial infection.^14,17^

Colorectal cancer (CRC) is one of the most prevalent and lethal forms of cancer throughout the world.^18^ Of the different histologic subtypes of CRC, adenocarcinoma is the most common and contains a distinct subtype referred to as mucinous adenocarcinoma, where 50% or more of the tumor volume is composed of mucinous components^19^ and is associated with significantly larger growth and increased lymph node infiltration.^20,21^ Unsurprisingly, this aggressive mucinous phenotype has been associated with a lower progression-free survival rate,^22^ especially in rectal cancer cases.^23^ Notably, mucinous adenocarcinoma is associated with increased *MUC2* expression^24-26^ and high MUC2 mucin expression is associated with colon cancer.^26^ Furthermore, the increased MUC2 mucin produced have a distinct glycan profile with a significant increase in fucosylation^27^ and a decrease in sialic acid.^28^ Similarly, IBD comprised ulcerative colitis (UC) and Crohn’s disease (CD) has also been intensively linked to MUC2 expression and mucus production.^29^ In UC patients, there is altered MUC2 secretion characterized by a thinner and more penetrable mucus layer,^30^ whereas in CD, there is an increase in mucus production.^31^ MUC2 mucin in UC is more sialylated, less sulfated, and overall less glycosylated as compared to healthy controls.^31^

MUC2 mucin glycans provide substantial substrate options for different microbes to bind through specific adhesin/ligand interactions and are degraded with glycan specific glycosidases. A normal microbiota is maintained by symbiotic microbes binding to the mucus layer, thereby preventing pathogenic organisms from colonizing the gastrointestinal mucus layer. However, pathogens may eventually gain access to the mucus layer and are well adapted in binding mucus. *Salmonella* Typhimurium has fimbriae that are specific for fucose residues in mucus^32,33^ as well as the ability to bind to sialic acid residues.^34^ *Vibrio cholera* uses flagella to propel itself through the mucus layer^1^ and has adhesins to bind fucose residues in the mucus layer.^35^ *Campylobacter jejuni* uses flagella to both migrate through the mucus layer and bind to terminal fucose residues in the mucus layer.^36^ Various *Escherichia coli* subtypes have different pili, fimbriae, or flagella that can bind to various sugars of MUC2.^1,35^ The colonic parasite *Entamoeba histolytica* uses an adhesin Gal/GalNAc lectin that binds to galactose and *N*-acetyl-galactosamine residues on colonic mucin, facilitating parasite colonization and eventual invasion.^37^ Similarly, *Yersinia enterocolitica* can bind to Gal/GalNAc residues on MUC2.^38^ Under healthy conditions, the mucus layer is sufficiently large and complex that the microbes and partially degraded mucus will be sloughed off and replaced by new mucus, constantly preventing invasion. However, if a microbe can degrade faster than the mucus layer can be replaced, the mucus layer may be breached, and the microbes can come into direct contact with the underlying epithelium. This can lead to invasion by pathogens, or inflammation as seen in diseases like IBD. In this study, we interrogated the functional properties of a high MUC2 mucin-producing goblet-like cell with mutations in various genes that affected MUC2 expression, cell proliferation, and tumorigenicity. These mutations collectively led to defective hypersecreted MUC2 mucus with alterations in glycomics profiles characterized by increased sialylated/fucosylated permeable mucus that rendered goblet cells highly susceptible to *Salmonella enterica* adherence, invasion, and cell death. These findings underscore how colonic adenocarcinoma mutations can dramatically shape and alter mucus barrier glycosylation and gut permeability in carcinogenesis.

## Results

### Mutant cells produce more MUC2 mucus than WT cells

**Figure 1.**
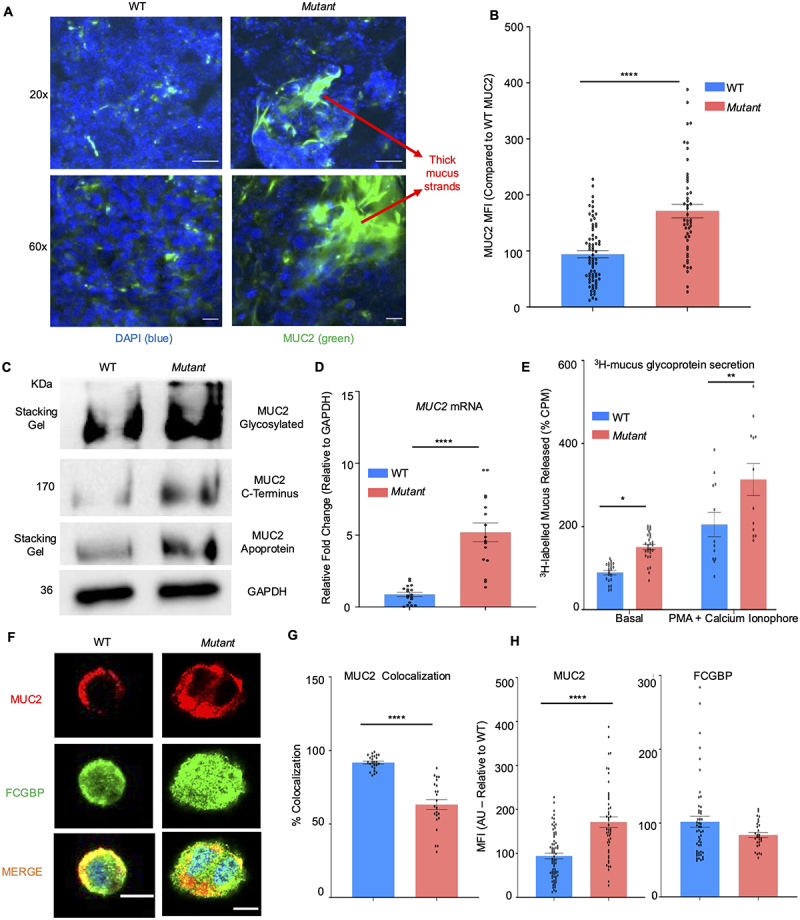
Increased MUC2 expression in hypersecretory *Mu*t cells *in vitro*. (A) Confocal microscopy images of WT and *Mut* cells showing MUC2 (green) and DAPI (blue). Note the thick mucus strands in *Mut* cells. Images are representative of three different experiments. Scale bar = 100 μm for 20x images and 20 μm for 60x images. (B) Quantification of mean fluorescent intensity (MFI) compared to WT cells for confocal images. ****p < 0.001. (C) MUC2 expression by Western blot in WT and *Mut* cells using antibodies specific for glycosylated MUC2 and the protein backbone of MUC2. (*n* = 5). (D) Quantification of *MUC2* mRNA expression in WT to *Mut* cells basally. (*n* = 15); ****p < 0.0001. (E) Quantification of newly secreted synthesized mucus glycoprotein from WT and *Mut* cells metabolically labeled with ^3^H-glucosamine basally and in response to the mucus secretagogues, PMA and calcium ionophore (10 μM). (*n* = 10–15); *p < 0.05, **p < 0.01. (F) Confocal microscopy images showing MUC2 (red), FCGBP (green), and DAPI (blue) in WT and *Mut* cells. Images are representative of five different experiments. Scale bar = 10 μm. (G) Quantification of MUC2 and FCGBP colocalization from the confocal images in F. ****p < 0.0001 (*n* = 15). (H) MFI of MUC2 and FCGBP as compared to WT cells from the confocal images in F (*n* = 15); ***p < 0.001.

As LS174T goblet-like cells are a mixed cell population with low-high MUC2 mucin producing phenotypes, we established an unbiased screen to isolate a high MUC2 mucus-producing clone though single cell limited dilutions of the parental WT goblet cell, similar to the characterization of mucin variants previously described.^39^ Based on mucus production in cell culture media and quantification by *MUC2* mRNA expression and Western blotting, a single cell colony was isolated and expanded as a hypersecretory cell line (henceforth referred to as the *Mutant* [*Mut*] cell line). A defining feature of the *Mut* cells in culture is that they constitutively secrete copious amounts of mucus strands that form dense clumps layered on the cells when stained and imaged with a MUC2 antibody by confocal microscopy (Figure 1A,B). Thus, to determine if *Mut* cells displayed altered biosynthesis and glycosylation of MUC2 basally, cell lysates were probed with antibodies specific for glycosylated MUC2, the *C*-terminus, and the apoprotein. All three antibodies showed constitutively enhanced MUC2 with a significant increase in *MUC2* mRNA expression as compared to WT cells (Figure 1C,D). To enumerate MUC2 mucin biosynthesis and secretion, WT and *Mut* cells were metabolically labeled with ^3^H-glucosamine that tags the newly synthesized mucus pool glycans^40^ to allow quantification of constitutive and mucus secretagogues-induced [phorbol 12-myristate^41^ (PMA) and calcium ionophore]^42^ mucus secretion. As predicted, *Mut* showed basally significantly more ^3^H-labeled mucin secretion than WT cells and enhanced secretion in response to the mucus secretagogues, PMA/calcium ionophore (Figure 1E). In addition to MUC2, the goblet cell specific protein FCGBP was also probed as it was recently shown to be biosynthesised, packaged, and released with MUC2 in mucin granules.^43^ As FCGBP and MUC2 are highly colocalized in WT goblet cells,^43^ confocal imaging using FCGBP- and MUC2-tagged antibodies was used to determine the distribution and intensity of both proteins in the cell. Interestingly, MUC2 was significantly less colocalized with FCGBP in *Mut* (~55% vs 90% in WT) cells despite the mutant having higher MUC2 mucin staining intensity (Figure 1F–H). The expression of FCGBP was similar in both cell types (Figure 1H). These data demonstrate that in *Mut* cells, MUC2 expression and secretion was enhanced but less colocalized with FCGBP as compared to WT cells.

### Whole genome sequencing of Mut cells revealed mutations in genes that affected MUC2 expression, cell proliferation, and differentiation

**Figure 2.**
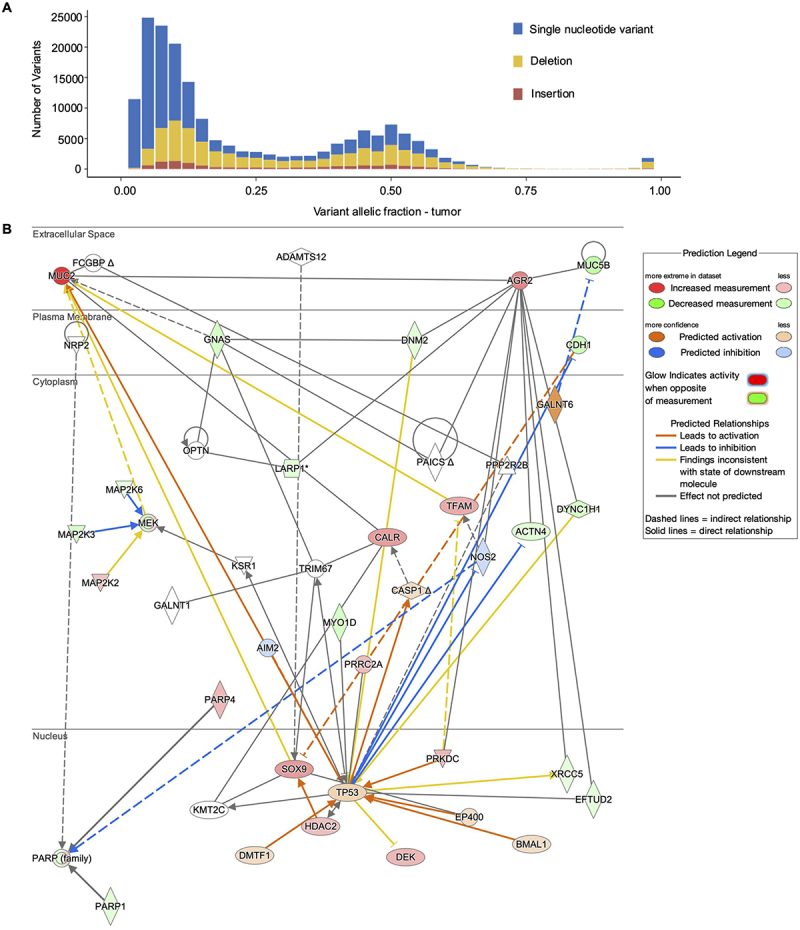
Whole genome sequencing of LS174T goblet-like *Mut* cells. (A) Frequency distribution of mutant alleles in the whole genome shotgun data. In a homogenous cell line, variant distribution is bimodal around 50% and 100%. (B) MUC2-centric (2 degrees) gene interaction diagram of all genes predicted as altered via whole genome sequencing, proteomic analysis, or PCR. Gene colors are based on up or down regulation according to proteomics.

To quantify mutations that were driving the high MUC2 mucin-producing phenotype, whole genome sequencing (WGS) was performed on WT and *Mut* cells. The frequency distribution of the mutations analyzed suggested a near-homogeneous colony of *Mut* cells, with most variant distribution being bimodal around 50% and 100% (Figure 2A). The summary statistics of the mutations in *Mut* as compared to WT cells are summarized by snpEff^44^ (Table 1). Of the 177,759 mutations identified, there were 104,921 single-nucleotide variants 11,853 insertions, and 60,985 deletions. While over 99% of these mutations were predicted to have no impact, a small percentage were considered to be of high, moderate, or low variant impact. The high impact variants (https://figshare.com/articles/dataset/Supplementary_Data_Set/28485530.), include frameshift or stop-gain variants (**Supplemental Table S1**). Integrated multi-omics analysis revealed an ensemble of mutations and protein-level perturbations that directly affected MUC2 gene regulation and cellular functions (https://figshare.com/articles/dataset/Supplementary_Data_Set/28485530.; **Supplemental Table S2**). In particular, one major pathway that was altered was related to TP53, a tumor suppressor gene associated with cell growth and division.^45^ Another major pathway identified was anterior gradient 2 (AGR2), which is fundamental in mucin biosynthesis and packaging in the endoplasmic reticulum.^46^ Mitogen-activated protein kinases (MEK and other MAPK) genes also created a major hub that affected MUC2 gene expression. MEK genes are associated with numerous essential cellular activities including cell proliferation and differentiation.^47^ The last major gene hub that was altered was the Poly [ADP-ribose] polymerase (PARP) family, including PARP1, which is associated with inflammation and apoptosis.^48^ Collectively, the WGS data showed numerous genes were mutated in *Mut* cells that directly affected MUC2, which could explain the increased MUC2 mucin-producing phenotype observed in *Mut* cells. Furthermore, several other pathways were altered that directly affected cell proliferation, inflammation, and protein synthesis, suggesting broad alterations affected *Mut* cellular functions.

**Table 1.** Key summary statistics of mutation effects from snpEff.

		Total	
Genome		GRCh38.mane.1.2.refseq	
Number of variants (before filter)		177,759	
Number of effects		189,740	
Genome effective length		3,088,762,395	
Variant rate		1 variant every 17,376 bases	
VARIANT TYPE	Total		
Single nucleotide variants	104,921		
Multiple nucleotide variants	0		
Insertions	11,853		
Deletions	60,985		
Inversions/Duplications/Translocations	0		
Total	177,759		
VARIANT IMPACT TYPE	Count		Percent
HIGH (frameshift and stop-gain variants)	225		0.12%
LOW (synonymous variants)	487		0.26%
MODERATE (missense variants and in-frame deletions)	671		0.35%
MODIFIER (untranslated and intergenic region variants)	188,167		99.27%
CODING VARIANT TYPE	Count		Percent
MISSENSE	655		67.18%
NONSENSE	29		2.97%
SILENT	291		29.85%

### Mut cells are upregulated in proteins related to stress and metabolism

**Figure 3.**
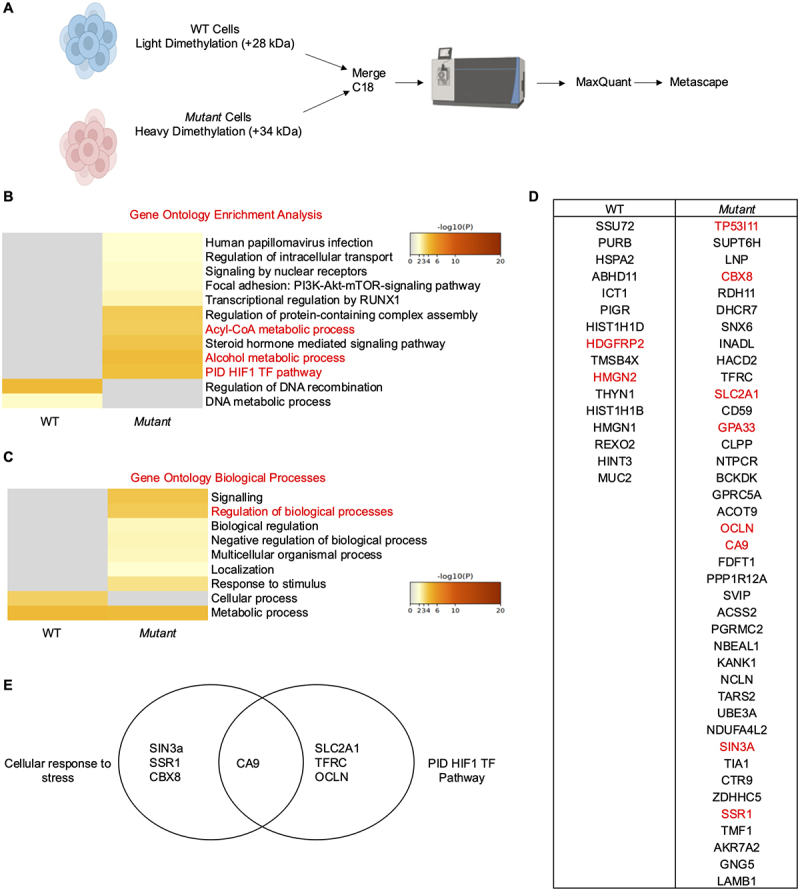
*Mut* cells are upregulated in genes related to stress and metabolism. (A) Workflow for proteomics analysis to compare WT and *Mut* cell lysates. (B) Metascape analysis showing gene ontology enrichment of pathways utilized by WT and *Mut* cells. Pathways of interest are highlighted in red. (C) Metascape analysis showing gene ontology biological processes associated with WT and *Mut* cells. Pathways of interest are highlighted in red. (C) Proteins that were upregulated in WT or *Mut* cells. Proteins of interest are highlighted in red. (D) Proteins from proteomic analysis that were significantly upregulated in WT or *Mut* cells. Proteins of interest are highlighted in red. (E) Venn diagram highlighting proteins of interest that were upregulated in *Mut* cells and part of the cellular response to stress, PID HIF1 TF pathway, or both.

To determine differences in the proteomes of *Mut* and WT cells, quantitative proteomic analysis was performed on whole cell lysates from both cell types as previously described.^49^ Briefly, WT cells were labeled with a light formaldehyde (+28 Da), while *Mut* cells were labeled with deuterated formaldehyde (+34 Da) (Figure 3A). Samples were combined, then run and analyzed simultaneously. Following liquid chromatography and tandem mass spectrometry analysis (LC-MS/MS), data was analyzed using MaxQuant^50^ and pathway analysis was performed using Metascape.^51^ A complete list and the relative quantification of proteins found in each cell type are listed in Table 2 and Supplementary Table S3 (https://figshare.com/articles/dataset/Supplementary_Data_Set/28485530). Gene ontology enrichment analysis revealed that *Mut* cells were upregulated in multiple pathways such as Acyl-CoA metabolic processes and alcohol metabolic process, whereas, WT cells were upregulated in pathways associated with regulation of DNA recombination and DNA metabolic process (Figure 3B). Of particular interest, regulation of intracellular transport and regulation of protein-containing complex assembly were both upregulated in *Mut* cells. These pathways could be related to increased MUC2 production and secretion (Figure 1) as mucin granule transport and assembly are required for proper MUC2 synthesis, storage, and exocytosis.^52^ Gene ontology of biological processes analysis revealed that *Mut* cells had an increase in genes associated with signaling regulation of biological processes, localization, and response to stimulus (Figure 3C). The proteins that were significantly upregulated in either WT or *Mut* cells were examined (Figure 3D). WT cells had significantly more Hepatoma-Derived Growth Factor-Related Protein 2 (HDGFRP2), a gene that is associated with repairing damaged DNA^53^. Similarly, WT cells were also upregulated for High Mobility Group Nucleosomal Binding Domain 2 (HMGN2), which exhibits antimicrobial activity that protects cells against various bacteria, viruses, and fungi^54^, and attenuates oxidative stress *in vitro*.^55^ *Mut* cells had increased expression of tumor protein p53 inducible protein 11 (TP53I11), a protein that is involved in cellular proliferation^56^ and is a transcriptional target of p53,^57^ which is itself altered in *Mut* cells by WGS (Figure 2B). Similarly, glycoprotein A33 (GPA33) was upregulated in *Mut* cells, and its upregulation is associated with colon cancer.^58^ Numerous proteins upregulated in *Mut* cells stood out due to their association with pathways associated with cellular response to stress: Signal Sequence Receptor Subunit 1 (SSR1), Chromobox homolog protein 8 (CBX8), SIN3 transcription regulator family member A (SIN3A), and Carbonic Anhydrase 9 (CA9), the latter of which is also associated with the PID HIF1 TF Pathway, which is associated with hypoxia inducible factors (HIF) (Figure 3E). The increase in stress-related and HIF pathways further demonstrate that the *Mut* cells are under high stress basally and are upregulated for numerous genes associated with stress and cancer, similar to what was revealed by WGS.

**Table 2.** Upregulated and downregulated proteins between WT and *mutant* LS174T cells found through proteomics. Proteins in black are upregulated in WT cells, whereas proteins in bold type are upregulated in *mutant* cells.

Gene Name	Protein name	Log2 (*Mut* to WT)
SSU72	RNA polymerase II subunit A C-terminal domain phosphatase SSU72	−2.963531833
PURB	Transcriptional activator protein Pur-beta	−2.641693769
HSPA2	Heat shock-related 70 kDa protein 2	−2.20769435
ABHD11	Alpha/beta hydrolase domain-containing protein 11	−2.162040616
ICT1	Peptidyl-tRNA hydrolase ICT1, mitochondrial	−2.158815851
PIGR	Polymeric immunoglobulin receptor;Secretory component	−2.150849399
HIST1H1D	Histone H1.3	−2.066910859
HDGFRP2	Hepatoma-derived growth factor-related protein 2	−2.040437548
TMSB4X	Thymosin beta-4; Hematopoietic system regulatory peptide	−1.995218186
HMGN2	Non-histone chromosomal protein HMG-17	−1.990738833
THYN1	Thymocyte nuclear protein 1	−1.882517721
HIST1H1B	Histone H1.5	−1.865542465
HMGN1	Non-histone chromosomal protein HMG-14	−1.829921119
REXO2	Oligoribonuclease, mitochondrial	−1.82863943
HINT3	Histidine triad nucleotide-binding protein 3	−1.828383229
MUC2	Mucin-2	−1.826386419
TP53I11	**Tumor protein p53-inducible protein 11**	**1.481712262**
SUPT6H	**Transcription elongation factor SPT6**	**1.504823754**
LNP	**Protein lunapark**	**1.524264315**
CBX8	**Chromobox protein homolog 8**	**1.525617899**
RDH11	**Retinol dehydrogenase 11**	**1.526970214**
DHCR7	**7-dehydrocholesterol reductase**	**1.594835149**
SNX6	**Sorting nexin-6; Sorting nexin-6, N-terminally processed**	**1.615369211**
INADL	**InaD-like protein**	**1.615981211**
HACD2	**Very-long-chain (3 R)-3-hydroxyacyl-CoA dehydratase 2**	**1.623024029**
TFRC	**Transferrin receptor protein 1; Transferrin receptor protein 1, serum form**	**1.625083418**
SLC2A1	**Solute carrier family 2, facilitated glucose transporter member 1**	**1.661977568**
CD59	**CD59 glycoprotein**	**1.690417096**
GPA33	**Cell surface A33 antigen**	**1.691221473**
CLPP	**ATP-dependent Clp protease proteolytic subunit**	**1.695370292**
NTPCR	**Cancer-related nucleoside-triphosphatase**	**1.700927932**
BCKDK	**[3-methyl-2-oxobutanoate dehydrogenase [lipoamide]] kinase, mitochondrial**	**1.724562965**
GPRC5A	**Retinoic acid-induced protein 3**	**1.75433169**
ACOT9	**Acyl-coenzyme A thioesterase 9, mitochondrial**	**1.767908995**
OCLN	**Occludin**	**1.774671112**
CA9	**Carbonic anhydrase 9**	**1.781359714**
FDFT1	**Squalene synthase**	**1.782198856**
PPP1R12A	**Protein phosphatase 1 regulatory subunit 12A**	**1.802193217**
SVIP	**Small VCP/p97-interacting protein**	**1.874836303**
ACSS2	**Acetyl-coenzyme A synthetase, cytoplasmic**	**1.90588998**
PGRMC2	**Membrane-associated progesterone receptor component 2**	**1.989175347**
NBEAL1	**Neurobeachin-like protein 1**	**2.004609253**
KANK1	**KN motif and ankyrin repeat domain-containing protein 1**	**2.006441658**
NCLN	**Nicalin**	**2.061465164**
TARS2	**Threonine – tRNA ligase, mitochondrial**	**2.40053793**
UBE3A	**Ubiquitin-protein ligase E3A**	**2.603311811**
NDUFA4L2	**NADH dehydrogenase [ubiquinone] 1 alpha subcomplex subunit 4-like 2**	**2.605494334**
SIN3A	**Paired amphipathic helix protein Sin3a**	**2.61223453**
TIA1	**Nucleolysin TIA-1 isoform p40**	**2.642193247**
CTR9	**RNA polymerase-associated protein CTR9 homolog**	**2.689120211**
ZDHHC5	**Palmitoyltransferase ZDHHC5; Palmitoyltransferase**	**2.830336463**
SSR1	**Translocon-associated protein subunit alpha**	**3.02313113**
TMF1	**TATA element modulatory factor**	**3.175269093**

### Stability of MUC2 mRNA and protein in Mut cells

**Figure 4.**
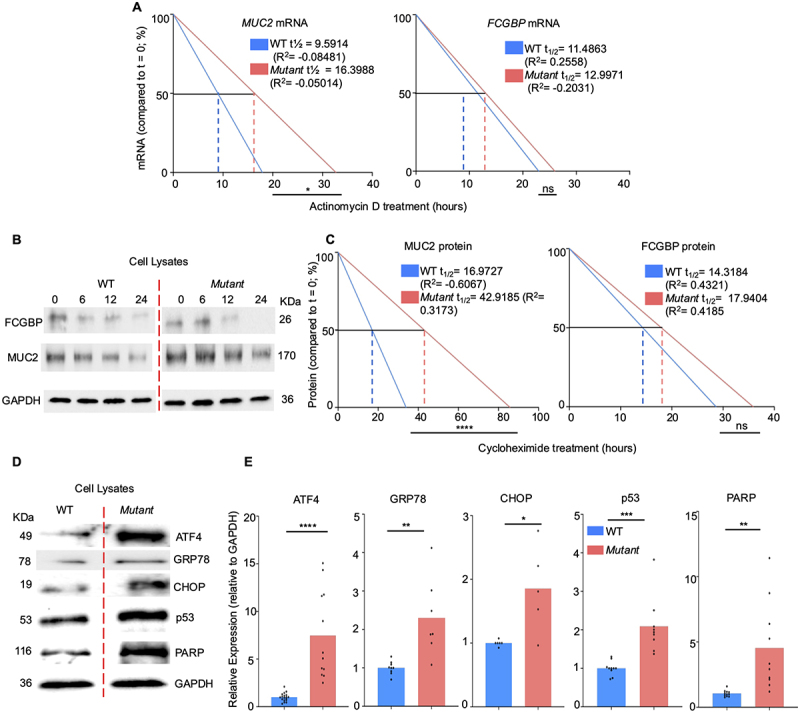
*Mut* cells are under more cellular stress than WT cells. (A) Regression analysis of *MUC2* and *FCGBP* mRNA in WT to *Mut* treated with the transcriptional inhibitor actinomycin D. mRNA was taken at 0, 0.5, 1, 2, 4, 6, 12, and 24 h post treatment with actinomycin D. The half-life is shown for each gene through regression analysis from mRNA quantified through RT-PCR. Data is from three independent experiments with three to five individual samples per experiment. Slopes were compared by analysis of covariance (ANCOVA, *n* = 9–15; *p < 0.05). (B) Western blot analysis of cell lysates from WT and *Mut* cells treated with the translational inhibitor cycloheximide. Protein was taken at 0, 6, 12, and 24 h post treatment with cycloheximide. Blots are representative of three independent experiments with two individual samples per experiment. (C) Regression analysis from Western blotting of MUC2 and FCGBP protein comparing WT to *Mut* treated with cycloheximide. The half-life is shown for each protein and was calculated by performing regression analysis on the densitometry analysis from the Western blots. Slopes were compared by analysis of covariance (ANCOVA, *n* = 6; ****p < 0.0001). (D) Basal expression of various stress proteins in WT and *Mut* cells. GAPDH was used as a housekeeping control. Blots are representative of 3–5 experiments. (E) Densitometry of Western blots of basal expression of various stress proteins normalized to GAPDH (*n* = 10–15); *p < 0.05, **p < 0.01, ***p < 0.001, ****p < 0.0001.

To unravel mechanistically why there was an increase in *MUC2* mRNA and protein expression in *Mut* cells, the stability of the MUC2 mRNA transcripts was determined. The stability of mRNA plays a major role in gene expression and the transient expression of protein. Accordingly, the half-lives of *MUC2* mRNA were quantified by RT-PCR in cells treated with the transcriptional inhibitor, actinomycin D.^59^ To interrogate if stability was specific to *MUC2*, the goblet cell specific protein FCGBP was also investigated as a goblet cell specific control. As predicted, *MUC2* mRNA showed significantly increased stability in *Mut* cells with a longer half-life (t½ 16.4 h in *Mut* vs t½ 9.6 h in WT, Figure 4A) that could account for higher MUC2 expression in *Mut* cells. *FCGBP* mRNA stability was unchanged between the cell types. To determine the half-lives of the MUC2 and FCGBP protein, cells were treated with the translation inhibitor cycloheximide^60,61^ and the fate of the protein was quantified at various times post treatment. Similar to mRNA stability, MUC2 protein half-life was significantly longer in *Mut* as compared to WT cells (t½ 43 h vs t½ 17 h in WT), whereas, the half-life for FCGBP was similar between the cell types (Figure 4B,C). These results demonstrate that in *Mut* cells, the half-life of MUC2 mRNA and protein were significantly more stable as compared to WT cells that could account for higher MUC2 protein expression. However, given that FCGBP was not as considerably different, this phenomenon seems unique to MUC2 and not to all goblet cell specific proteins.

As high MUC2 mucin production is associated with endoplasmic reticulum (ER) stress,^62–64^ and proteomics analysis revealed numerous pathways associated with stress in *Mut* cells, we next measured basal expression of several stress proteins enumerated by Western blotting (Figure 4D,E) and they were significantly upregulated in *Mut* as compared to WT cells. These include the stress protein activating transcription 4 (ATF4), a transcription factor that is induced by stress and upregulated in certain cancers.^65^ In addition, the overexpression of glucose regulating protein 78 (GRP78), C/EBP homologous protein (CHOP), and Poly (ADP-ribose) polymerase (PARP) are all associated with ER stress and apoptotic pathways. ^48,66–68^ The tumor associated protein p53 was also markedly upregulated, indicative of cellular stress that could induce apoptosis.^69^ Of note, both PARP and TP53 were major hubs identified through WGS (Figure 2B) to be affected by mutations in the *Mut* cell line. The significant increase in the stress proteins noted in *Mut* cells predict that biochemical processes and metabolic pathways would be severely altered in these cells.

### Mut cells display an altered glycomics profile

**Figure 5.**
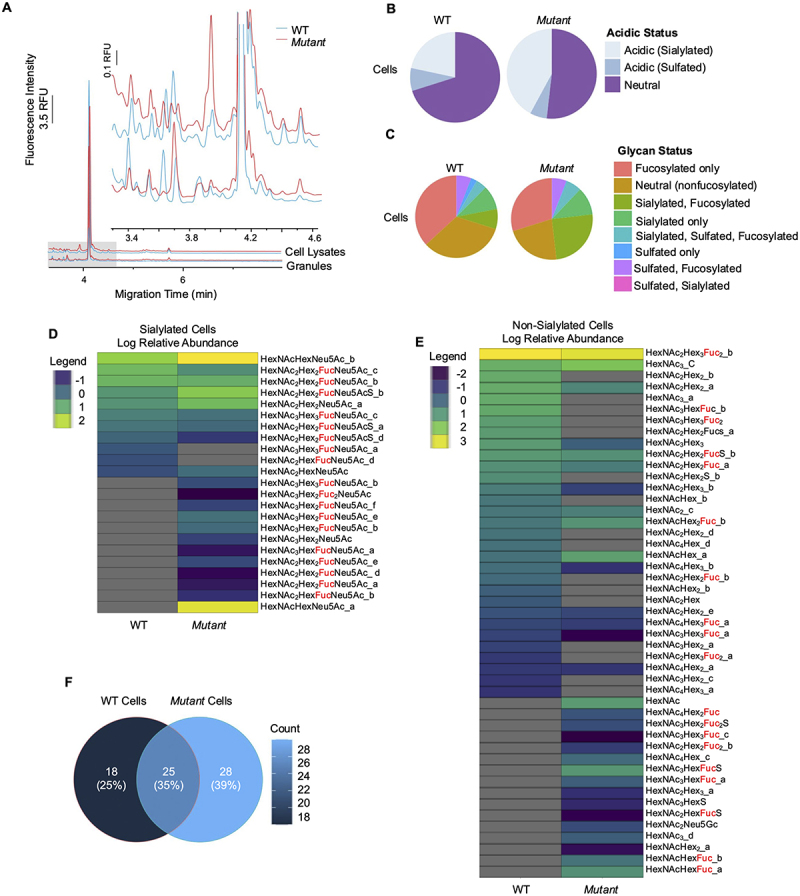
*Mut* cells display an altered glycomics profile than WT cells. (A) CE-LIF analysis of WT and *Mut* cell lysates and granules. (B). Pie chart of acidic (sialylated or sulfated) vs. neutral glycans for WT and *Mut* cell lysates. (C) Pie chart of relative abundance of glycan classes for WT and *Mut* cell lysates. (D) Heatmap of log2-transformed relative abundances of individual sialylated glycans found in WT and *Mut* cell lysates. Glycans with the same composition but unique isobars are indicated with an underscore and number. Fucosylated glycans are marked in red. Grey indicates the glycan was not present. (E) Heatmap of log2-transformed relative abundances of individual non-sialylated glycans found in WT and *Mut* cell lysates. Glycans with the same composition but unique isobars are indicated with an underscore and number. Fucosylated glycans are marked in red. Grey indicates the glycan was not present. (F) Venn diagram of overlapping and unique glycan structures identified in WT and *Mut* cells. Glycan numbers are indicated and the percentage of total analyzed is indicated in parentheses.

To define if the glycans that comprised the mucus from WT and *Mut* cells were altered, sensitive glycomic analyses were performed on cell lysates and purified mucin granules by both capillary electrophoresis with laser-induced fluorescence detection (CE-LIF; Figure 5A) and high-performance liquid chromatography-mass spectrometry (HPLC-MS), the latter technique employing either ammonium formate^70,71^ (Figure 5B–D) or formic acid^72^ (**Supplementary Figure S1, Supplementary Table S4;** https://figshare.com/articles/dataset/Supplementary_Data_Set/28485530) as HPLC mobile phase additives to achieve the resolution of glycans using a porous graphitic carbon (PGC) stationary phase. While acidic phases are commonly used in MS research, basic (*i.e*. ammonium formate-containing) mobile phases have been demonstrated to enhance the ionization of glycans, in particular sulfated species, allowing for a more complete analysis.^73^ CE-LIF (Figure 5A) provided a rapid, quantitative overview of the *O*-linked glycome of both WT and *Mut* cells as well as their specialized secretory granules (discussed below). Electropherograms were dominated by a large peak at 4.1 min; however, there were noticeable differences in the glycomic profiles, particularly between 3.4 and 4.6 min, suggestive of subtle differences in the glycome of these cells. Since CE-LIF does not yield compositional information in the absence of standards (few of which are available for *O*-glycans), HPLC-MS was used to more clearly define the glycomic differences between these cells (Figure 5B–E).

The overall differences between WT and *Mut* cells were compared by analyzing the proportion of glycans that were sialylated or sulfated. WT cells were mostly neutral (containing no fucose, sialic acid, or sulfation) with some sialylated or sulfated glycans (Figure 5B). By contrast, *Mut* cells had an overall higher level of acidic glycans, specifically with an expansion in sialylated glycans. To further discern differences between glycans, the samples were again grouped based on the presence or absence of fucose, sialic acid, or sulfur residues. As seen previously, *Mut* cells had less neutral glycans and instead had a higher proportion of sialylated residues as well as sialylated residues that were also fucosylated and/or sulfated (Figure 5C). To further interrogate the glycan differences between cell types, all sialylated glycans found in either cell type were plotted on a heat map (Figure 5D). There was a clear and distinct increase in sialylated residues that were found solely in *Mut* cells. Of note, several of the sialylated glycans found only in *Mut* cells were also fucosylated (red). All non-sialylated glycans found in cells were also plotted in a heat map, and again, fucosylated glycans were marked in red (Figure 5E). There was a marked increase in fucosylated glycans found only in *Mut* cells. Together, this demonstrated that *Mut* cells have a unique glycan population made of significantly sialylated, and to a lesser extent, fucosylated glycan residues. The unique and overlapping glycans between cells, granules, and both groups were compared via Venn diagrams (Figure 5F). Thirty-five percent of the glycans found in cell samples were found in both WT and *Mut*, whereas 25% and 39% were unique to WT and *Mut* cells.

For completeness, the data set obtained from the acidic mobile phase chromatography was also analyzed and likewise, demonstrated that WT and *Mut* cells had unique glycomic profiles. The vast increase in sialylated glycans, as well as glycans that were both sialylated and fucosylated were again noted in this analysis (**Supplementary Figure S1A-D**). Of note, less glycans were found in this analysis than through the ammonium formate analysis, and less glycans were found in *Mut* than WT cells overall (**Supplementary Figure S1E**). However, the general trends for increased sialylation and fucosylation were found in both analyses in *Mut* cells. Taken together, these results demonstrate that *Mut* cells have a unique quality and quantity of glycans with a marked increase in sialylated glycans.

**Figure 6.**
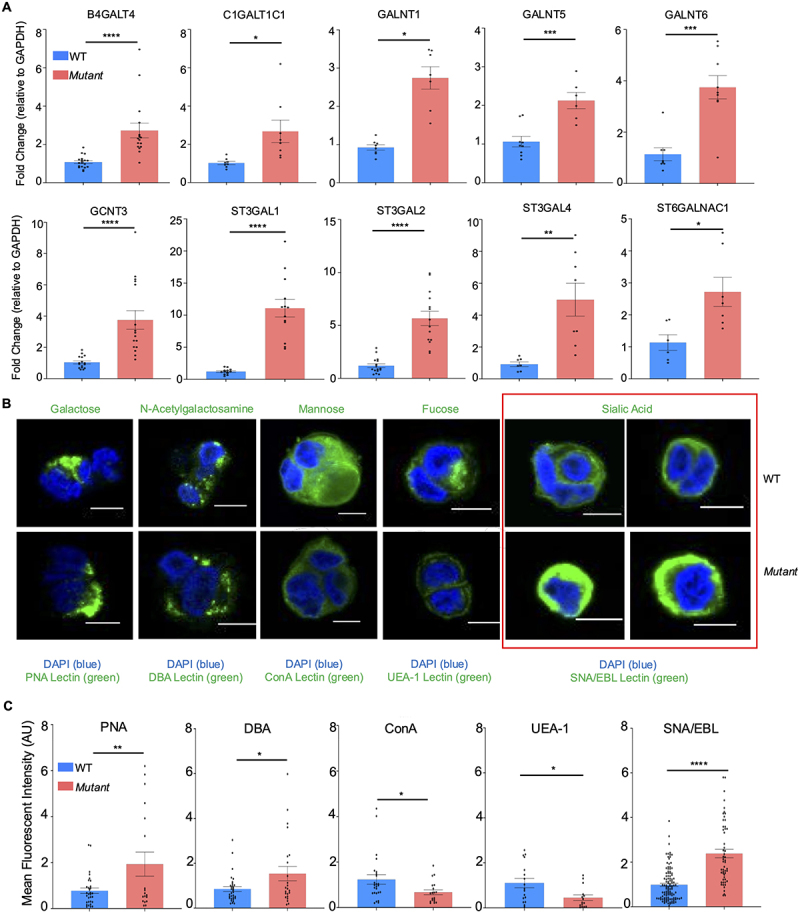
*Mut* cells are more sialylated that WT cells. (A) RT-PCR of glycosyltransferases that are upregulated in *Mut* cells (*n* = 8–12); *p < 0.05, **p < 0.01, ***p < 0.001, ****p < 0.0001. (B) Confocal microscopy of lectin-specific glycans (green) in WT and *Mut* cells with DAPI (blue). The lectin used were peanut agglutin (PNA) for galactose, *dolichos biflorus* agglutinin (DBA) for N-acetylgalactosamine, concanavalin a (ConA) for mannose, *ulex europaeus* agglutinin (UEA-1) for fucose, and *Sambucus nigra* (elderberry bark) SNA/EBL for sialic acid. Images are representative of three independent experiments. Scale bar = 10 µm. (C) Quantification of lectin specific glycans imaged by confocal microscopy. *p < 0.05, **p < 0.01, ****p < 0.0001.

To corroborate these results, we determined if there was a corresponding increase in the expression of various glycosyltransferases by quantitative PCR in WT and *Mut* cells. The galactosyltransferase *B4GALT4* was upregulated in *Mut* cells, as was the galactosyltransferase chaperone *C1GALT1C1* (Figure 6A). Additionally, *N*-acetylgalactosaminyltransferases *GALNT1*, *GALNT5*, and *GALNT6* and the *N*-acetylglucosaminyltransferase *GCNT3* were also significantly upregulated. As predicted, the sialyltransferases were significantly upregulated, including *ST3GAL1*, *ST3GAL2*, *ST3GAL4*, and *ST6GALNAC1*. The increase in sialyltransferases could explain the increase in sialylation observed in *Mut* cells by glycomics analysis. To enumerate the cell surface glycans, fluorescently labeled lectins were used on WT and *Mut* cells and visualized by confocal microscopy. The neutral non-reducing residues galactose and *N*-acetylgalactosamine, visualized by peanut agglutin (PNA) and *Dolichos biflorus* agglutinin (DBA), respectively, were significantly upregulated in *Mu*t cells, whereas mannose, imaged by Concanavalin A (ConA), was upregulated in WT cells (Figure 6B,C). The variety of the staining patterns with the different neutral lectins further highlights the unique glycan profiles for each cell type. α1,2-linked fucose residues, visualized with *Ulex europaeus* agglutinin I (UEA-1), were upregulated in WT cells, consistent with the HPLC-MS glycomics data collected with the acidic mobile phase (**Supplementary Figure S1B, C**). Finally, α2,6-linked sialic acid, imaged by *Sambucus nigra* (elderberry bark) lectin (SNA/EBL), was strikingly increased in *Mut* cell, consistent with both HPLC-MS glycomics data sets, as well as the increased sialyltransferase expression. Taken together, these results demonstrate that sialylation was markedly increased in *Mut* as compared to WT cells.

### Mut cells produce high reactive oxygen species basally that drives sialylation in the cell

**Figure 7.**
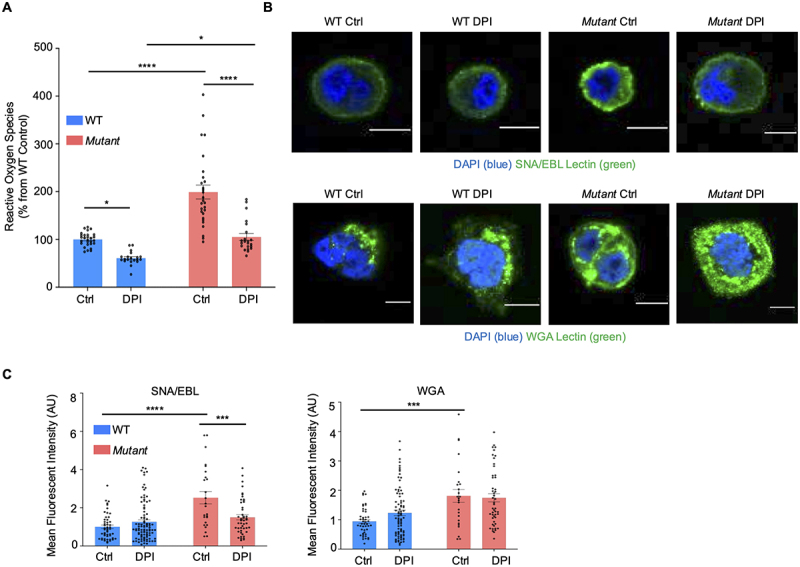
Increased ROS production drives increase sialylation in *Mut* cells. (A) Reactive oxygen species (ROS) quantification in WT and *Mut* cells basally and in response to overnight treatment with the ROS inhibitor DPI. Experiment was repeated three independent times (*n* = 14–20); *p < 0.05, ****p < 0.0001. (B) Confocal microscopy of the neutral lectins with wheat germ agglutinin (WGA) and for N‐acetylglucosamine and sialylated lectins with *Sambucus nigra* (elderberry bark) (SNA/EBL). Cells were imaged basally or after overnight treatment with the ROS inhibitor DPI. Images are representative of three independent experiments. Scale bar = 10 µm. (C) Quantification of lectin specific glycans imaged by confocal microscopy with and without DPI (*n* = 14–20); ***p < 0.001, ****p < 0.0001.

As *Mut* cells are under considerable ER stress with high expression of ATF4, GRP78, CHOP, PARP, and p53 as compared to WT cells (Figure 4D), we hypothesized that reactive oxygen species (ROS) were at least, in part, responsible for the excessive sialylation. Previous studies have shown that oxidative stress in particular, was associated with increase sialic acid expression.^74^ As predicted, basally *Mut* produced significantly more ROS than WT cells (Figure 7A) and treatment with the ROS inhibitor diphenyleneiodonium (DPI) significantly reduced ROS in both cell types. Of note, DPI treatment inhibited ROS produced by *Mut* cells to basal levels produced by WT cells. To interrogate if the reduction in ROS affected glycosylation in the cells, DPI-treated cells were stained with SNA/EBL, and DPI had no effect on sialic acid expression in WT but significantly decreased SNA/EBL expression in *Mut* cells (Figure 7B,C). To specifically determine that DPI was altering sialic acid and not all glycans, wheat germ agglutinin (WGA) was used as a control to visualize primarily *N*-acetylglucosamine. Basally, *Mut* showed significantly more WGA staining than WT cells, and the addition of DPI did not alter the expression of WGA in either cell type (Figure 7B,C), suggesting that the reduction in ROS specifically affected sialic acids. Collectively, these data suggest that the increased sialylation in *Mut* cells was mechanistically driven by high levels of ROS that reverted to a WT phenotype by inhibiting ROS.

### Mucin granules isolated from Mut cells display an altered glycomics profile

**Figure 8.**
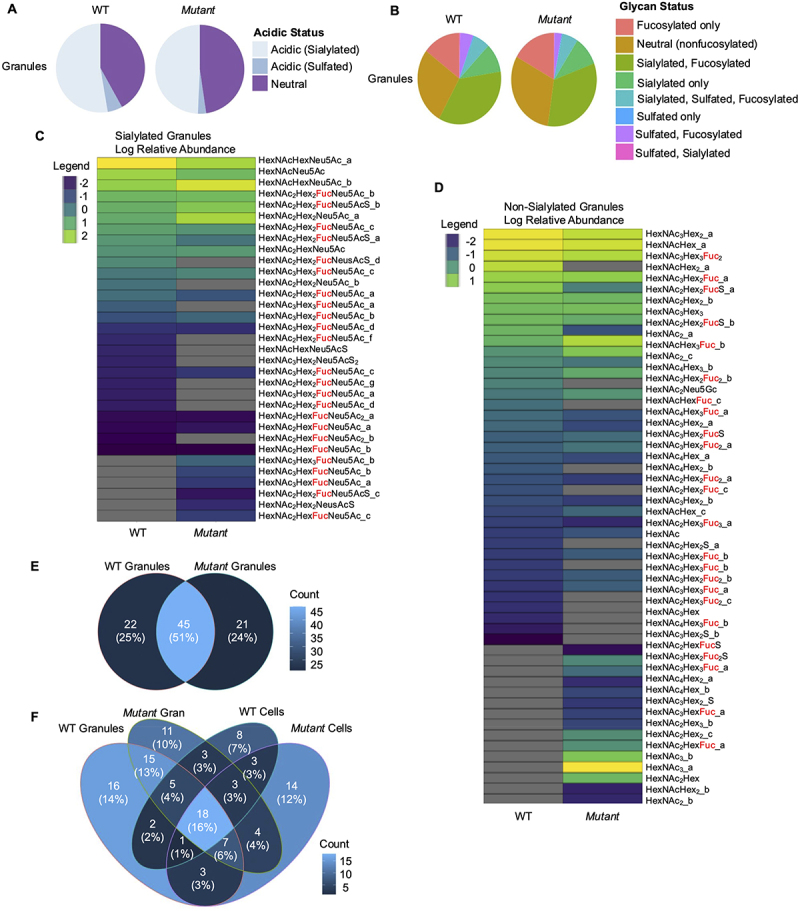
*Mut* derived mucin granules display an altered glycomics profile as compared to WT granules. (A). Pie chart of acidic (sialylated or sulfated) vs. neutral glycans for WT and *Mut* granules. (B) Pie chart of relative abundance of glycan classes for WT and *Mut* granules. (C) Heatmap of log2-transformed relative abundances of individual sialylated glycans found in WT and *Mut* granules. Glycans with the same composition but unique isobars are indicated with an underscore and number. Fucosylated glycans are marked in red. Grey indicates the glycan was not present. (D) Heatmap of log2-transformed relative abundances of individual non-sialylated glycans found in WT and *Mut* cell lysates. Glycans with the same composition but unique isobars are indicated with an underscore and number. Fucosylated glycans are marked in red. Grey indicates the glycan was not present. (E) Venn diagram of overlapping and unique glycan structures identified in WT and *Mut* granules. Glycan numbers are indicated, and the percentage of total analyzed is indicated in parentheses. (F) Venn diagram of overlapping and unique glycan structures identified in WT and *Mut* cells and granules. Glycan numbers are indicated, and the percentage of total analyzed is indicated in parentheses.

As *Mut* basally have a different glycomic profile than WT cells, we hypothesized that MUC2 mucin granules of each cell type would also be unique. MUC2 mucin and mucus associated proteins are highly expressed in mucin granules, and the granules are important packaging and storage vesicles for mucin exocytosis. As with whole cell lysates, MUC2 mucin granules were purified, and then sensitive glycomics analyses were performed by CE-LIF (Figure 5A) and HPLC-MS using an ammonium formate mobile phase (Figure 8) or formic acid-containing mobile phase (**Supplementary Figure S1**). The glycans that were found in each granule type were graphed based on acidic status and showed that WT and *Mut* derived granules had a slightly different glycomics profile (Figure 8A). Like before, the glycans were categorized as neutral (containing no fucose, sialic acid, or sulfation), fucosylated, sialylated, or sulfated. Unlike cell lysates, *Mut* granules had a higher composition of neutral and fucosylated glycans as compared to WT granules (Figure 8B). As with the cells, the sialylated and non-sialylated glycans found within granules of WT or *Mut* cells were plotted on heat maps (Figure 8C,D). Again, the differences in glycans between genotypes was more subtle, but demonstrated a slight tendency for the *Mut* granules to be more fucosylated. A Venn diagram was prepared to show the differences between the amount of glycans found in each granules type. The WT and *Mut* granules both contained 51% of the glycans found, and each granule type had 25% or 24% unique glycans (Figure 8E). A Venn diagram was also prepared to contrast the glycans found between genotypes in both cells and granules (Figure 8F). Interestingly, 18 glycans were found in all samples regardless of the location of the genotype.

The HPLC separation of glycans using the acidic mobile phase also confirmed a unique glyco-profile between the granule types (**Supplementary Figure S1**). The acidic phase analysis demonstrated an increase in fucosylated glycans (**Supplementary Figure S1A, B**). Of note, the majority of glycans in *Mut* granules were common to WT granules as well, suggesting a loss of non-fucosylated glycans in *Mut* granules (**Supplementary Figure S1F-H**). These data demonstrate that not only cell metabolism and physiology were altered in *Mut* cells but that the biosynthesis and glycosylation of MUC2 mucin granules were also affected.

### Mut cells are more tumorigenic than WT cells

**Figure 9.**
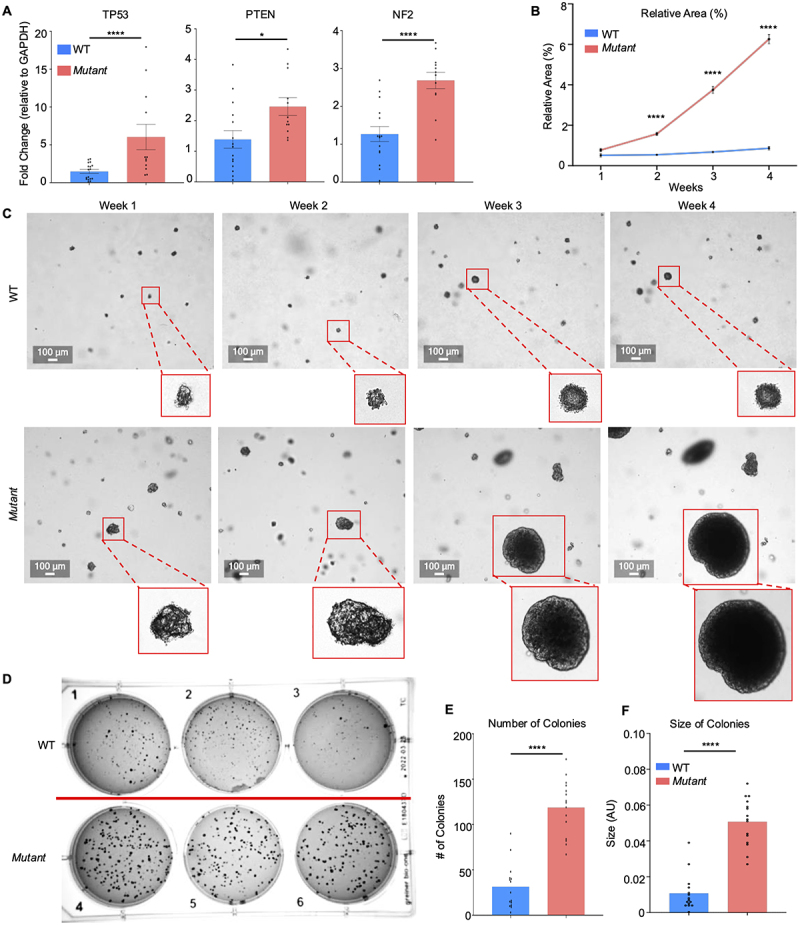
*Mut* cells are more tumorigenic than WT cells. (A) Quantification of various tumor associated gene mRNA expression in WT and *Mut* cells basally. (*n* = 15); *p < 0.05, ****p < 0.0001. (B) Relative area of each well that was covered by a tumor over 4 weeks. Images were taken weekly on a CellCyte X imager. 18 wells of each cell type were imaged weekly with 36 images taken per well. (C) Representative images of weekly tumor growth by WT and *Mut* derived tumors taken on the CellCyte X imager. (D) Representative images of tumor growth after 4 weeks. Tumors were stained with crystal violet to visualize the growth. *n* = 18. (E) Number of distinct tumor colonies counted in each cell type after 4 weeks of growth. ****p < 0.0001. (F) Size of tumor colonies after 4 weeks of growth. ****p < 0.0001.

Since high cellular stress is associated with cancer,^75,76^ and WGS showed numerous genes associated with the cancer marker TP53 are altered (Figure 2A), we determined if stressed *Mut* cells were more tumorigenic. First, we identified eight oncogenic or potentially oncogenic mutations from the whole genome sequencing using the Personal Cancer Genome Reporttool^77^ (Table 3). Next, we quantified the expression of various cancer-associated genes TP53,^45^ PTEN,^78^ and NF2,^79^ and they were all significantly increased in *Mut* as compared to WT cells (Figure 9A). To enumerate tumor growth, WT and *Mut* cells were grown in soft agarose into tumor-like spheres.^79^ This assay is the most stringent for monitoring anchorage-independent growth for detecting tumorigenicity. The colonies were measured weekly for 4 weeks to determine growth and microscopic images taken to follow tumor progression. During weeks 2 through 4, *Mut* derived tumors grew exponentially and were significantly larger and covered more surface area than WT-derived tumors (Figure 9B,C). Whereas WT-derived tumors grew steadily from week to week, *Mut* derived tumors grew exponentially creating large spheroids. After week 4, the tumor colonies were stained with crystal violet and imaged, revealing significantly more and larger *Mut* tumors (Figure 9D–F). These data demonstrate that *Mut* cells expressed significantly more cancer-associated genes and were highly tumorigenic as compared to WT cells.

**Table 3.** Analysis of oncogenic phenotype I *Mut* cells as performed using the personal cancer genome reporttool.

Symbol	Protein Change	Gene name	Consequence	Oncogenicity
CDH1	p.Leu355Ter	cadherin 1	Frameshift	Oncogenic
KMT2C	p.Ser2984PhefsTer18	lysine methyltransferase 2C	Frameshift	Oncogenic
EPAS1	p.Gln561SerfsTer7	endothelial PAS domain protein 1	Frameshift	Oncogenic
HNF1A	p.Pro291ArgfsTer25	HNF1 homeobox A	Frameshift	Oncogenic
FAT4	p.Gln4186Ter	FAT atypical cadherin 4	Frameshift	Oncogenic
SPEN	p.Ala2251GlnfsTer102	spen family transcriptional repressor	Stop gained	Oncogenic
DAB2IP	p.Leu746SerfsTer47	DAB2 interacting protein	Frameshift	Oncogenic
DAB2IP	p.His1025ProfsTer4	DAB2 interacting protein	Frameshift	Likely oncogenic

### Mut cells secreted mucus are more penetrable to fluorescent microspheres (beads) and Salmonella enterica invasion

**Figure 10.**
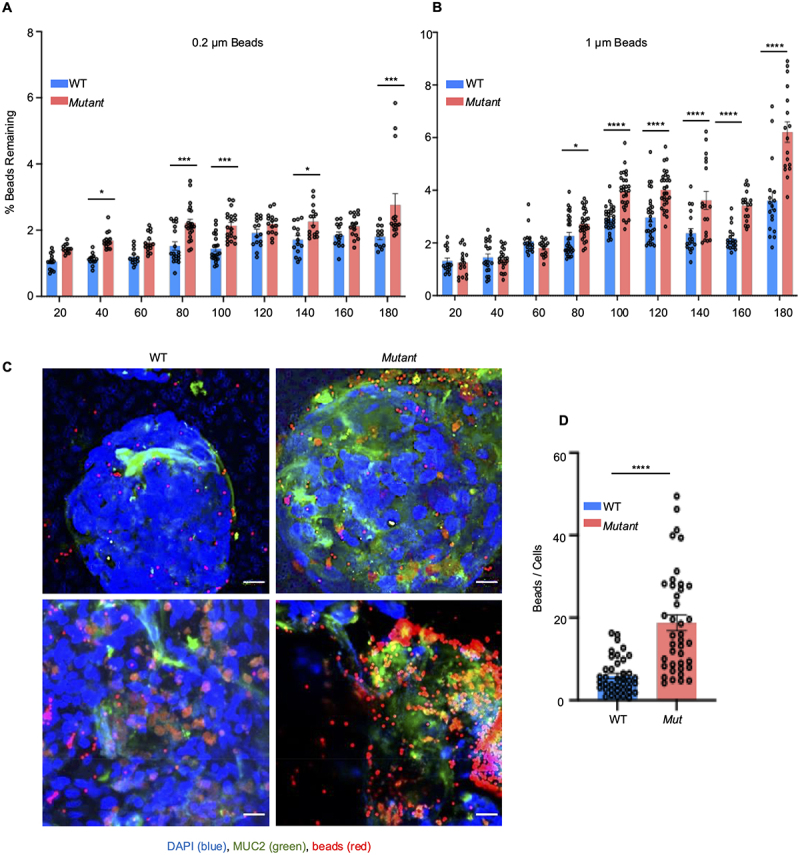
Hypersecretory *Mut* cells are more penetrable by microbeads. (A) 0.2 µM bead or (B) 1 µM bead penetrability in WT and *Mut* cells. Experiment was repeated three independent times (*n* = 10–15). *p < 0.05, ***p < 0.001, ****p < 0.0001. (C) Confocal microscopy of 1 µM fluorescent beads on WT and *Mut* cells. Scale bar = 20 μm. (D) Quantification of 1 µM beads that penetrated after 60 min imaged by confocal microscopy (scale bar = 20 µm). *n* = 10–15; ****p < 0.0001.

To determine if the *Mut* cells secreted mucus was more penetrable, fluorescent carboxylate-modified microspheres (0.2 μm and 1 μm) were layered on top of the secreted mucus produced by WT and *Mut* cells for various time points. Different sizes of beads were used to represent small toxins or viral particles and bacteria.^30,80,81^ Following incubation, the media and unbound beads were removed, and the remaining beads were enumerated by fluorescence. The mucus layer was presumed to be more penetrable if more beads got embedded in the mucus layer. As predicted, significantly more 0.2 μm beads penetrated the mucus layer from *Mut* cells as early as 40 min and plateaued for up to 180 min (Figure 10A). Interestingly, 1 μm beads were significantly more penetrable than 0.2 μm beads after 80–180 min post inoculation (Figure 10B) as compared to WT. To visualize penetration of the beads, 1 μm beads were inoculated for 60 min on coverslips that showed significantly more beads penetrated *Mut* cell mucus as compared to WT cells (Figure 10C,D). Collectively, these data demonstrated that *Mut* cell secreted mucus was dysfunctional by its inability to exclude the penetration of 0.2 and 1 μm microspheres.

**Figure 11.**
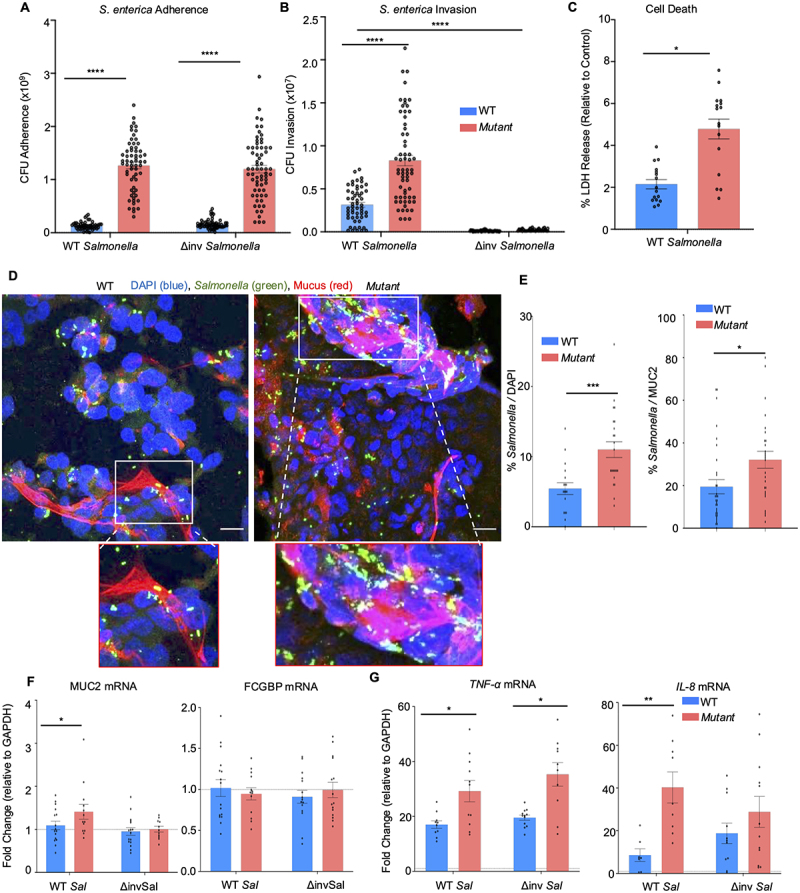
*Salmonella enterica* adherence and invasion enhanced cells death in *Mut* cells. As controls, both WT and a less invasive *S. enterica* strain that has a kanamycin insertion mutation in the invasion gene a (δinv) was used. (A) *S. enterica* adherence and (B) invasion after 1 h in WT and *Mut* cells. Experiment was repeated five independent times (*n* = 20–30); ****p < 0.0001. (C) Cell death was quantified by LDH release from WT and *Mut* cells in response to *S. enterica* invasion after 2 h. Experiment was repeated five independent times (*n* = 12–15); *p < 0.05. (D) Confocal microscopy of *S. enterica* adherence on WT and *Mut* cells at 1 h showing MUC2 (red), *S. enterica* (green), and DAPI (blue). Images are representative of three different experiments. Scale bar = 20 μm. Inset is a close-up view of *salmonella* (green) bound to mucus strands (red). (E) Quantification of *S. enterica* adherence to cells and mucus by confocal microscopy (*n* = 12–15); *p < 0.05, ***p < 0.001. (F) RT-PCR analysis of MUC2 and FCGBP in WT and *Mut* cells in response to *S. enterica* at 1 h. Dotted lines indicate control values (*n* = 12–15); *p < 0.05. (G) PCR analysis of the pro-inflammatory cytokines TNF-α and IL-8 in WT and *Mut* cells in response to *S. enterica* after 1 h. Dotted line indicates control values (*n* = 12–15); *p < 0.05, **p < 0.01.

To determine if the increased bead penetration observed in *Mut* cells depicts a more penetrable mucus layer to potentially enhanced invasion by a live pathogen, WT and *Mut* cells were inoculated with live *Salmonella enterica*, and adherence, and invasion were measured after 1 h. As a control for invasion, a noninvasive strain of *S. enterica* (Δinv *Salmonella*) was used. Even though WT and Δinv *Salmonella* significantly adhered to *Mut* cells (Figure 11A), only WT *S. enterica* invaded the mutant cell (Figure 11B) associated with high cell death as quantified by lactate dehydrogenase (LDH) release (Figure 11C). To visualize bacteria adherence, *S. enterica* cultures were fluorescently labeled and showed enhanced clumping of bacteria adherent on the mucus strands (insets) in *Mut* as compared to WT cells (Figure 11D,E). While the above study quantified the functional properties of secreted mucus to *S. enterica* infection, it was also of interest to determine the cellular innate host defense responses between the cells during invasion. In response to *S. enterica* infection after 1 h, *MUC2* mRNA expression was significantly upregulated in *Mut* but not WT cells; there was no change in response to Δinv *Salmonella* (Figure 11F). *FCGBP* transcripts were not altered in either WT or *Mut* cells in response to *Salmonella*, suggesting the increase in *MUC2* is specific to the mucin gene and not due to increased global goblet cell product transcription. However, both WT *S. enterica* and Δinv *S. enterica* significantly upregulated the expression of the pro-inflammatory cytokines *TNF-α* and *IL-8* in *Mut* as compared to WT cells (Figure 11G). Other pro-inflammatory cytokine genes (*IL-1ß* and *IL-6*) were not upregulated. These data demonstrate that even though *Mut* cells secreted more mucus and mounted a stronger pro-inflammatory response, they were more susceptible to *S. enterica* adherence, invasion, and cell death.

### Wounded Mut cells healed faster than WT

**Figure 12.**
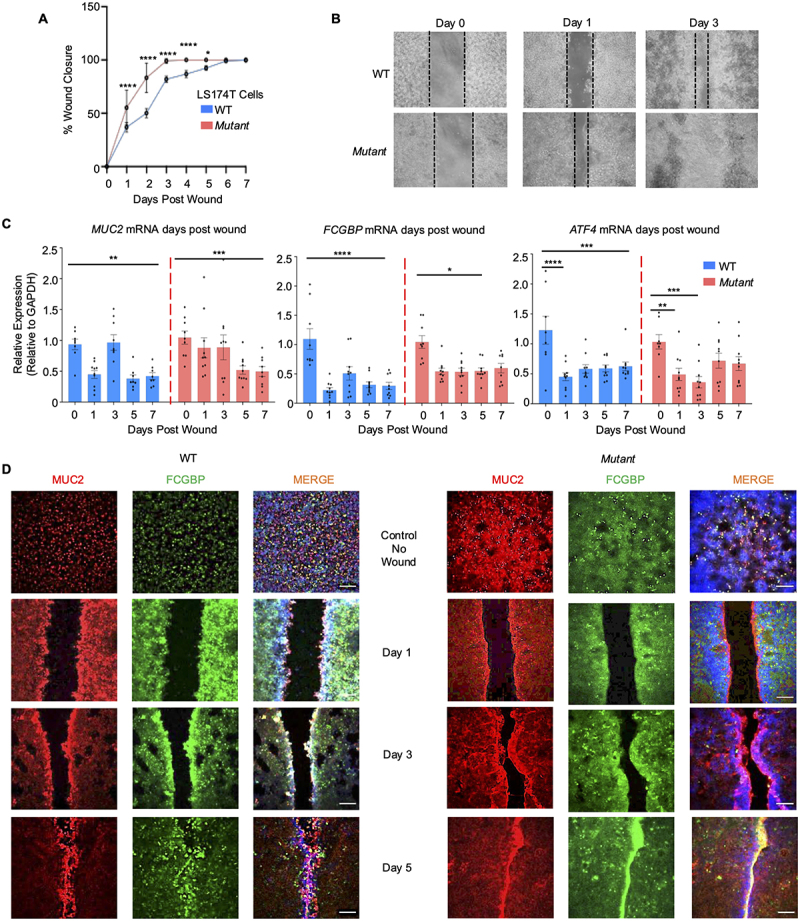
*Mut* cells display faster restitution than WT goblet cells. (A) WT and *Mut* cells were grown with a wound insert, leaving a gap of 500 μm. At 0 to 7 d post wound, the space between the cells was measured to quantify restitution (*n* = 6 per day); *p < 0.05, ****p < 0.0001. (B) Representative bright field images of the restitution assay. The solid black dotted lines represent the cell front during wound healing. (C) At 0 to 7 d post wound, *MUC2*, *FCGBP*, and *ATF4* mRNA were analyzed by RT-PCR. GAPDH was used as a housekeeping gene (*n* = 6 per day); *p < 0.05, **p < 0.01, ***p < 0.001, ****p < 0.0001. (D) Confocal microscopy images of control WT and *Mut* during restitution showing MUC2 (red), FCGBP (green), and DAPI (blue). Scale bar = 200 μm. Images are representative of three different experiments.

We have previously shown that high MUC2 mucin expression in goblet cells was associated with increase ER stress and impaired wound healing.^63^ More recently,^43^ we advanced this observation to show that it was the colocalization of FCGBP with MUC2 that impeded wound closure at the margin of wounds. Based on these findings, we theorize that *Mut* cells that produce high levels of MUC2 but do not colocalize with FCGBP (Figure 2F,H) would abrogate this effect. To characterize the rate and pattern of wound healing in *Mut* compared to WT cells, wound inserts were used to mechanically injure the cells, and restitution was followed temporally. When the wound margin was measured, *Mut* cells had significantly smaller gaps as early as 1-d post insert removal and were completely healed by day 3 (Figure 12A,B). In WT cells, wound closure was gradual with complete closure by day 5 (Figure 12A). *MUC2* and *FCGBP* mRNA were not altered during wound closure in both cell types as compared to their respective controls (Figure 12C). In contrast, the levels of the stress marker *ATF4* was downregulated as early as day 1 and remained low in WT cells. However, in *Mut* cells, *ATF4* mRNA expression was slightly increased on days 5 and 7 post wound. Given that ATF4 is associated with cell migration and wound healing *in vitro*,^82^ it is plausible that this increase could be indicative of wound closure. In addition, the release of wound healing growth factors measured by a multiplex assay showed that *Mut* cells produced significantly higher levels of EGF,^83^ HB-EGF,^84^ and IL-8^85^ as compared to WT cells (**Supplementary Figure S2A**). By confocal microscopy, WT wounds showed high expression of colocalized MUC2 and FCGBP at the wound margin as the wound closed on day 5 (Figure 12D, Supplementary Figure S2B, C). In contrast, MUC2 and FCGBP were not colocalized in *Mut* cells to the wound margin that healed within 3 days (Figure 12D, Supplementary Figure S2C). The appearance of FCGBP was evident when the wound was fully healed on day 5. Finally, migration and proliferation were measured in WT and *Mut* cells to determine if either or both processes were responsible for the increased wound healing observed in *Mut* cells. Indeed, both migration, as measured by paxillin^86^, and proliferation, as measured by 5-ethynyl-2’-deoxyuridine (EdU)^87^ incorporation, were significantly increased in *Mut* cells (**Supplementary Figure S2D, E**). Collectively, these data demonstrate that the hypersecretory MUC2 producing phenotype in *Mut* cells accelerated wound healing by increased secretion of growth factors, disassociation of FCGBP from MUC2 mucin and with higher cell migration and proliferation.

## Discussion

To our knowledge, this is the first study to characterize by multi omics naturally occurring mutations in goblet-like cells that affected *MUC2* mRNA expression, mucus hyperproduction, and alterations in glycomics profiles associated with increased tumorigenicity. In WT goblet cells, FCGBP and MUC2 are highly colocalized^43^ and in *Mut* cells uncoupling of FCGBP with MUC2 mucin weakened the mucus protective barrier that rendered cells susceptible to *S. enterica* adherence, invasion, and cell death. Surprisingly, increased ER stress in *Mut* cells upregulated growth factor production that accelerated wound healing and cancer-associated gene expression that enhanced tumorigenicity. These findings underscore the multifunctional roles of MUC2 in the mucus barrier as an essential component of innate host defense, but also to prevent cellular metabolic stress to avoid inappropriate proliferation and aggressive tumor development.

The increased MUC2 expression in *Mut* cells was evident through various quantification methods including RT-PCR, Western blotting and confocal imaging for protein, and ^3^H-glucosamine labeled mucus secretion. The initial discovery of a hyper secreted mucus phenotype led us to hypothesize that perhaps the *Mut* cells produced a stronger mucus barrier that was less susceptible to pathogen invasion. Surprisingly, we saw a significant increase in *S. enterica* adhesion, invasion, and cell cytotoxicity in *Mut* cells despite the increase in constitutive MUC2 mucus secretion. These findings highlight that MUC2 quantity alone is insufficient to form a proper mucus layer under homeostatic conditions. In addition to the upregulation in MUC2 mucin, *Mut* cells also showed upregulated ER stress proteins. This was not surprising given that previous reports have observed that when compared to either *MUC2KO* cells or low mucin producing cells, high MUC2 producing WT cells had higher ER stress and ROS production, leading to increased apoptosis and autophagy.^62,64^ In comparison, *Mut* produced even higher MUC2 and ROS production than WT cells. Based on these findings, we speculate that the increased stress in *Mut* cells could cause various metabolic disorders in an animal or human with an increased mucus phenotype due to any number of genetic mutations. Upregulated ER stress is associated with numerous inflammatory diseases including atherosclerosis, cardiomyopathy, liver fibrosis, IBD, diabetes mellitus, Parkinson's disease, and Alzheimer's disease,^88^ among others.

We have previously interrogated the role of ER stress, wound healing, and autophagy in relation to MUC2.^62–64^ Increased MUC2 production was associated with increased ER stress due to the heavy demand of mucus glycosylation in the goblet cell, decreased wound healing due to the high ER stress, and increased autophagy as the cell removes misfolded protein to maintain homeostasis. Constitutively activated autophagy in a mouse model was shown to enhance the mucus barrier,^89^ suggesting a positive correlation between autophagy and MUC2 mucus production. In particular, protection from dextran sodium sulfate (DSS) and infectious AIEC-induced colitis was observed when mucus was fortified through constitutively activated autophagy; more mucus resulted in more protection. While this seems intuitive, this was not what we observed in our model. More mucus from the *Mut* cells did not result in protection against microbead penetration or *Salmonella* invasion and cell death. The difference may lie within the mucus phenotype; the altered glycomics seems to be a key mediator in the mucus, providing protection. Thus, mucus quality and quantity are important in proper mucosal protection. Another study also described a damaged mucus layer caused by antibiotic-induced ER stress.^90^ This correlates with our current model where increased ER stress seems to be associated with a weakened, dysfunctional mucus layer.

While it is evident that *Mut* cells have a higher MUC2 phenotype, this was not apparent by proteomics. Glycoproteins, including mucins such as MUC2, are notoriously challenging to correctly identify and analyze through proteomics as many glycosylated peptides have a different mass that the predicted amino sequence. MUC2 mucins are immensely decorated by a dense layer of *O-*glycosylated glycans, which covers the protein core, preventing it from being properly identified by proteomic equipment.^91^ Appropriately, samples were processed according to a protocol designed to accommodate *O-*glycosylation.^92^ However, it remains plausible that MUC2 remained partially hidden by glycosylation, skewing the proteomic results. It is also plausible that different types of glycans are more susceptible to the proteomic pre-treatment. Given that WT and *Mut* cells had vastly different glycomic profiles, it is possible that the level to which they were sufficiently reduced differed, giving rise to the contradictory MUC2 quantities detected. Conversely, the other methods of MUC2 detection that were used were independent of glycosylation, and therefore, it remains more than likely that the abundant evidence supporting *Mut* cells having higher MUC2 expression remains true.

Whole genome sequencing uncovered several mutations in genes that affected MUC2 mucin biosynthesis, glycosylation, and production in *Mut* cells. Numerous genes were associated with *TP53*, which itself was upregulated in *Mut* cells as shown by RT-PCR. It was recently shown that p53 transcriptionally activated MUC2 in numerous cell types including colonic cells.^93^ While it is well understood that too little or no mucus can be associated with colon cancer,^94^ it was also shown that increased mucus production can be associated with worse cancer outcomes.^95^ In addition to genes related to TP53, AGR2 was also upregulated as shown by proteomics. A deficiency in AGR2 has been demonstrated to lead to a decrease in MUC2 mucin formation,^96^ as AGR2 is essential in MUC2 synthesis and goblet cell proliferation.^46,97^ Furthermore, AGR2 induction has been associated with mucin overproduction in the airways in asthma.^98^ Thus, it is not surprising to observe AGR2 upregulation in *Mut* cells. A final interesting connection to MUC2 came from genomics analysis, which showed that the protein phosphatase 2 regulatory subunit Bbeta (PPP2R2B) gene was disrupted in *Mut* as compared to WT cells. Downregulation of PPP2R2B has recently been associated with worsened clinical outcomes in breast cancer^99^ and inactivation of PPP2R2B is associated with CRC. Of interest, PPPP2R2B is predicted to interact with the key goblet cell protein FCGBP, which in turn is known to interact with MUC2.^43,100^ This provides yet another line of evidence suggesting numerous genes are altered in *Mut* cells that explains the hypersecretory mucus phenotype and increased tumorigenicity. Future studies could aim to knockdown or overexpress key mutations that were identified to be driving mucus hypersecretion such as PARP, TP53, AGR2, and MAPK. It would be mechanistically insightful to determine if alterations in any of these genes alone could recapitulate the phenotype seen in the *Mut* cell line. However, based on the data presented, our study argues against a single dominant mutation that was driving MUC2 hypersecretion and alterations in glycomics profiles and cellular functions. More than likely, mucus hypersecretion was driven by a combination of mutations in various genes associated with MUC2 that had profound effects on overall goblet cell functionality.

The altered glycomics profile identified in *Mut* cells and mucin granules was a major finding in this study. Altered glycosylation is unequivocally associated with colorectal cancer progression/manifestation^101–103^ and IBD, with more severe alterations associated with worsened disease symptoms and outcomes in UC. In particular, *C1GALT1C1* has been identified as a risk gene in IBD,^104^ and *C1GALT1C1* mRNA expression was upregulated in *Mut* cells. Increased sialylation in *Mut* cells has also been reported in association with UC and CD patients.^105^ These studies highlight the delicate balance of glycosylation required to maintain homeostasis and avoid colonic inflammation caused by improper glycosylation. Furthermore, as MUC2 expression is already used as a prognosis marker for colorectal cancer,^106,107^ this highlights the need to understand the glycomic differences associated with normal and pathogenic mucus layers.

MUC2 is a well-established colorectal cancer marker,^106^ and high production of mucus is associated with mucinous adenocarcinoma.^22^ Indeed, a previous study investigated sub clones of LS174T cells and found that high mucus production was also associated with tumorigenicity in mice.^39^ Therefore, it was not surprising to observe increased tumorigenicity from the hypersecretory *Mut* cells, which is itself a cancer cell line derived from a colorectal adenocarcinoma. In *Mut* cells, there were eight mutations related to seven genes that were described as oncogenic or likely oncogenic. Of these, *CDH1*, the gene encoding E-cadherin, was of interest due to previous studies suggesting E-cadherin may be involved in gastrointestinal cancers^108^ and CD.^109^ Further investigation revealed that mutations in *CDH1* are associated with various gastrointestinal cancers including the stomach and large intestine where mucus is highly prevalent.^110^ Additionally, mutations in *EPAS1* were also noted to be oncogenic, and aberrant activation of *EPAS1* has been associated with mucus hypersecretion.^111^ *HNF1A* has recently been nominated as a gene that regulated colorectal cancer progression in certain patients,^112^ although a link to mucus is currently unclear. These observations suggest that our cell line model could recapitulate common, well-defined oncogenic events and serve as a useful tool for interrogating mucus hypersecretion in cancer progression, especially for the highly tumorigenic mucinous phenotypes.

The penetrability of small microbeads has previously been used to assess the quality of the mucus layer.^30,81^ Studies have shown that mice with induced colitis or patients with active UC have a more penetrable mucus layer as demonstrated by an increase in microbead penetration. In this study, we showed that the mucus layer in *Mut* cells was more penetrable, similar to the mucus layer of active UC patients. Interestingly, other findings by the same group found a decrease in the goblet cell protein FCGBP in patients with active UC.^92^ We have recently demonstrated that FCGBP and MUC2 are closely spatially organized in secreted mucus,^43^ and we speculate that altering this delicate association could affect the stability of the entire mucus network, as seen in *Mut* cells. The increased penetrability of inert microbeads mirrored the data on invasion of *S. enterica* where more bacteria adhered to and invade the cells. We were not surprised to observe increased S. *enterica* adhesion and invasion in *Mut* cells that stimulated the pro-inflammatory cytokine TNF-α and the chemokine IL-8, both of which can initiate and exacerbate inflammation.^113^ It was also recently shown that high mucus production from goblet cells is associated with higher production of IL-8.^114^ An impaired mucus layer with increased bacterial invasion and cell death is common in IBD.^115^ MUC2 glycosylation confers protection against and susceptibility to pathogenic invasion in the gut. In this study, we used *S. enterica* as a model pathogen in part due to previous studies highlighting the interaction between *S. enterica* with specific glycan components.^32-34^ *S. enterica* contains fibril adhesins in its Std operon that bind with high affinity to fucosylated glycans on colonic mucus.^32,33^ Importantly, *Mut* granules contained more fucosylated glycans; thus, the secreted mucus layer derived from these granules with high fucose residues would account for increased *S. enterica* binding to *Mut* mucus. Other studies have highlighted that *S. enterica* can readily bind to sialic acids,^34^ which were markedly upregulated in *Mut* cells. Furthermore, higher concentrations of free fucose and sialic acid derived from the mucus layer have been associated with higher degrees of expansion of pathogens including *S. enterica, Clostridium difficile*, ^116^ and *E. coli*. ^117,118^ *Helicobacter pylori* is also reported to have increased adherence in the presence of fucose and sialic acid residues in the stomach,^119–122^ and although our study focuses on colonic mucus, it is reasonable to assume that similar alterations would occur in other regions of the gastrointestinal tract with increased susceptibility to various gastrointestinal pathogens.

An interesting discovery was that of the *Mut* cells, which showed increased migration and proliferation, with high levels of growth factors leading to faster wound healing. These findings were similar to previous studies^43,63^ comparing WT and *MUC2KO* goblet cells, where *MUC2KO* cells healed significantly faster. The faster healing was associated with the absence of MUC2 colocalized with FCGBP. Here, the same phenomenon was observed using a different model; there was faster healing in the *Mut* cells when there was significantly less colocalization of MUC2 and FCGBP. It is becoming clear that if either MUC2 or FCGBP is missing or altered, wound healing seems to be augmented. It is unclear if MUC2 and FCGBP colocalization at a wound site is disadvantageous, or if the cells are upregulating growth factors and proliferation as a compensatory mechanism. Either way, it is noteworthy to observe increased wound healing in the absence^43^ or hypersecretion of MUC2, given that most evidence in this and previous studies describe negative outcomes if MUC2 is downregulated or missing.^93-95^

In summary, the data presented in this study provide innovative insights into the currently unclear consequence of hyper secreted MUC2 mucus in various models of disease. Here, we uncover mutations by whole genome sequencing associated with MUC2 that led to increased oxidative stress with enhanced MUC2 secretion with an altered glycomics profile characterized by high levels of fucose and sialic acid residues. This led to hyper secreted mucus that was more penetrable to microspheres and *S. enterica* bacterial adhesion and invasion, which could be risk factors in numerous gastrointestinal inflammatory diseases like IBD.

## Materials and methods

**Manuscript Preparation**: All authors had access to the study data and had reviewed and approved the final manuscript.

### Human goblet cells

WT and *Mut* cells from the human colorectal adenocarcinoma cell line LS174T were maintained in a humidified incubator with 5% CO_2_. Cells were grown in complete Eagle’s Minimum Essential Medium (EMEM) supplemented with heat inactivated fetal bovine serum (10%), HEPES (10 mM), penicillin (100 U/mL), and streptomycin (100 µg/mL). Cells were passaged weekly, and media was changed every 2 to 3 d.

The *Mut* cell was produced using an unbiased screen to isolate a high MUC2 mucin producing clone by single cell limited dilution of the parental WT cells. A similar approach was used to produce a high mucin variant of LS174T cells to determine differences in the phenotypes associated with constitutive mucus secretion^39^. In total, we screened over 200 single cell clones that showed visible mucus strands in culture. From these, we selected five clones that showed the highest mucus producing phenotype as quantified by increased *MUC2* mRNA expression and protein secretion by Western blotting. Following sub cloning of the 5 clones over 10 passages in culture, we subsequently selected one *Mut* clone for the study that exhibited the highest mucus producing phenotype as quantified by copious amount of mucus strands in culture and with the highest expression of *MUC2* mRNA expression and protein secretion (Figure 1).

### Confocal microscopy

LS174T cells were grown overnight on No. 1.5 glass coverslips as previously described^43^. Briefly, coverslips were washed in PBS, fixed with paraformaldehyde (4%) for 15 min at 37°C, washed with PBS, and permeabilized with 0.35% Triton for 5 min. After three washes with PBST (0.1% Tween-20 in PBS), coverslips were blocked for 1 h with donkey serum (5%), washed, and incubated overnight with antibodies for MUC2 (sc -515,106, Santa Cruz) or FCGBP (PAP389Hu01, Cloud-Clone) at 4°C in a humidified chamber. The next day, PBST was used to wash the coverslips prior to incubating with fluorescent secondary antibodies and DAPI (Life Technologies) for 1 h. Lectins were imaged with PNA (FL-1071, Vector Laboratories), DBA (FL-1031, Vector Laboratories), ConA (FL-1001, Vector Laboratories), SNA/EBL (FL-1301, Vector Laboratories), UEA-1 (FL-1061, Vector Laboratories), and WGA (FL-1021, Vector Laboratories). For migration and proliferation studies, cells were counterstained with Paxillin (05–417, Millipore) or with EdU incorporation measured as per the manufacturer’s instructions (C10637, Thermo Fisher). FluorSave (Calbriochem) was used to mount the coverslips. Imaging was performed on a Nikon A1R confocal laser scanning microscope. Fiji (ImageJ) was used for quantification of protein intensity and colocalization.

### Immunoblotting

Protein lysates from LS174T cells were collected, washed with PBS, and lysed with lysis buffer containing EDTA (1 mmol/L), Tris-HCl (20 mmol/L), SDS (0.1%), NaCl (100 mmol/L), Triton X-100 (0.5%), phenylmethylsulfonyl fluoride (PMSF), and protease inhibitor cocktail (Sigma-Aldrich) on ice for 30 min. Samples were then centrifuged at 8,000 x *g* for 10 min to remove the pelleted debris. Protein was quantified with a BCA protein assay, and equal concentrations of protein were resuspended in 5X Laemmli buffer with β-mercaptoethanol and boiled for 5 min. To determine the half-life of proteins, cells were incubated with 10 μg/mL of cycloheximide (C7698; Sigma Aldrich) for 0, 6, 12, or 24 h prior to protein extraction and Western blot analysis. Regression analysis was performed on the densitometry of the Western blots to determine the half-life of the protein. For supernatant from cells, spent media were collected and centrifuged at 1,500 x *g* for 5 min to remove any cells or debris and subsequently concentrated through trichloroacetic acid (TCA) precipitation prior to being resuspended in 5X Laemmli buffer with β-mercaptoethanol and boiled for 5 min. The resulting samples were loaded into SDS polyacrylamide gels and then transferred onto a 0.22 μm nitrocellulose membrane. After transferring, the membranes were blocked in skim milk powder (5%) in PBST for 1 h. The membranes were then probed with primary antibodies overnight; MUC2 (in house antibody against CsCl purified glycosylated MUC2 mucin,^40^ Santa Cruz sc -515,106 for the C-terminus, or Santa Cruz sc-7314 for the apoprotein), FCGBP (PAP389Hu01, Cloud-Clone), ATF4 (11815, Cell Signaling), GRP78 (3177, Cell Signaling), CHOP (2895, Cell Signaling), PARP (9542, Cell Signaling), and p53 (sc-126, Santa Cruz). Mouse monoclonal anti-glyceraldehyde 3-phospate dehydrogenase (GAPDH) (Calbiochem 6C5) was used as a housekeeping protein. Corresponding HRP-conjugated secondary antibodies were diluted in PBST with 3% skim milk powder and subsequently visualized using Immobilon Western Chemiluminescent HRP Substrate (Sigma Millipore) and imaged on a Bio-Rad ChemiDoc Imager.

### Quantitative real-time PCR

RNA from cells was extracted using the Omega Bio-Tek E.Z.N.A. Total RNA Kit, as per the manufacturer’s instructions, and the RNA yield was measured using a NanoDrop1000 spectrophotometer. qScript cDNA SuperMix (QuantaBio) was used to reverse transcribe 500 ng of RNA. qPCR was performed using the following primers and annealing temperatures: Human MUC2 (F: CCTGGCCCTGTCTTTGG, R: CTTCAGGTGCACAGCAAATTC, 60°), Human FCGBP (F: GGACCTCAAGAACACTGGCA, R: GAGGATGGAGACTGAAGCGG, 60°), Human ATF4 (F: AGTTCGACTTGGATGCCCTG, R: CCAACGTGGTCAGAAGGTCA, 56°), Human IL-1β (F: GCTCTGGGATTCTCTTCAGC, R: TGGTGGTCGGAGATTCGTAG, 60°), Human TNF-α (F: AAGCCTGTAGCCCATGTTGT, R: GAGGTACAGGCCCTCTGATG, 65°), Human IL-8 (F: CTGGCCGTGGCTCTCTTG, R: CCTTGGCAAAACTGCACCTT, 60°), Human TP53 (F: CCTCAGCATCTTATCCGAGTGG, R: TGGATGGTGGTACAGTCAGAGC, 56°), Human PTEN (F: TGAGTTCCCTCAGCCGTTACCT, R: GAGGTTTCCTCTGGTCCT-GGTA, 62°), Human NF2 (F: CACATCCAGTCAGAAACCAGTGG, R: GGAATGTCTGCG-CCAAAAGCTG, 62°), Human GAPDH (F: TGATGACATCAAGAAGGTGGTGAAG, R: TCCTTGGAGGCCATGTGGGCCAT, 60°). Reactions were done in a Corbett Rotor Gene 3000 system. Fold change was quantified using the 2^−ΔΔCT^ method. For experiments determining the half-life of mRNA, 5 μg of Actinomycin D (11421; Cayman Chemicals) was used for 0, 0.5, 1, 2, 4, 6, 12, and 24 h prior to mRNA extraction and RT-PCR analysis. Regression analysis was performed on the resultant RT-PCR analysis to determine the half-life. Glycosyltransferases were screened by RT-PCR using a QuantStudio™ 3 Real-Time PCR System (Thermo Fisher) using plates and primers for Human B4GALT1, C1GALT1C1, GALNT1, GALNT5, GALNT6, GCNT3, ST3GAL1, ST3GAL2, ST3GAL4, ST6GALNAC1, and GAPDH designed by Bio-Rad.

### Quantification of mucus secretion

Mucins from WT and *Mut* cells were metabolically labeled with 2 µCi/mL of ^3^H-glucosamine as previously described.^123^ After 48 h, cells were washed thrice with serum-free EMEM and 10 μM PMA and 10 μM calcium ionophore were added to wells for 2 h to induce MUC2 mucin secretion in a total volume of 500 μL per well. Two hundred and fifty microliter of the supernatant containing ^3^H-secreted mucin was loaded into a scintillation vial for measuring ^3^H-activity in a scintillation counter (Beckman Coulter).

### Whole genome sequencing

Sequencing libraries for the wildtype and mutant cells were generated using the PCR-free DNA library prep kit with tagmentation from Illumina (Carlsbad, CA). 2 × 150 bp Illumina NGS sequencing was performed using a P4 XLEAP flow cell on a NextSeq2000 instrument, to a depth of 77x and 76x for the wildtype and mutant, respectively. Mutations in the mutant cell line were identified using the Dragen 4.2 (Illumina, Carlsbad, CA) paired somatic-germline DNA variants pipeline, including analysis of small nucleotide variants, structural variants, and copy number variants. High impact mutations were identified using snpEff^44^ version 5.1, namely frameshift and stop-gain variants. Transcript database GRCh38.mane.1.2.refseq was used for making these effect predictions. Analysis of oncogenic phenotype was performed using the Personal Cancer Genome Reporttool^77^ version 2.0. The whole dataset was uploaded to NCBI under the accession number PRJNA1166286.

### Shotgun proteomics analysis

WT and *Mutant* LS174T goblet cells were used for shotgun proteomics analysis as previously described in detail.^43^ Cell lysis (1% SDS, 0.1 M EDTA in 200 mM (pH 8)) and protease inhibitor tablets (Roche) were done on the samples before the samples were reduced with 10 mM DTT. Alkylation was done using 15 mM iodoacetamide in the dark for 25 min at room temperature. Dimethylation reaction was done for 18 h at 37°C using heavy (40 mM ^13^CD_2_O +20 mM NaBH_3_CN [sodium cyanoborohydride]) or light (40 mM light formaldehyde [CH_2_O] + 20 mM NaBH_3_CN) formaldehyde. Next, samples were desalted using C18 chromatography before high-performance liquid chromatography and tandem mass spectrometry (MS/MS).

### Mass spectrometry (MS)

All MS/MS proteomics experiments were carried out by the Southern Alberta Mass Spectrometry (SAMS) core facility at the University of Calgary, Canada, as previously described.^49^ Analysis was performed on an Orbitrap Fusion Lumos Tribrid MS (Thermo Scientific) operated with Xcalibur (version 4.0.21.10). Two microgram of tryptic peptides were added onto a C18 trap at a flow rate of 2 μl/min of solvent A (0.1% formic acid and 3% acetonitrile in HPLC-MS grade water). Elutions of peptides were done at a 120 min gradient from 5% to 40% (5–28% in 105 min followed by an increase to 40% B in 15 min) of solvent B (0.1% formic acid in 80% HPLC-MS grade acetonitrile) at a flow rate of 0.3 μL/min and separated on a C18 analytical column (75 μm × 50 cm; PepMapRSLC C18; P/N ES803; Thermo Scientific). Peptides were then electrosprayed on the Orbitrap Lumos operating in positive mode. The Orbitrap first performed a full MS scan at a resolution of 120,000 FWHM to detect the precursor ion having a *m*/*z* between 375 and 1,575 and a + 2 to + 7 charge.

### Proteomic data and bioinformatics analysis

Spectra were matched to peptide sequences of the human UniProt protein database using MaxQuant as done previously.^124-126^ Search parameters were set as Trypsin/P, with up to two missed cleavages. The data were analyzed using Metascape^51^ for reactome analysis to generate the enrichment pathway.

### Glycan sample preparation

Whole cell lysates or granules were transferred into 1.5 mL screw-capped centrifuge tubes (Sarstedt) for HPLC-MS analysis as previously described.^127^ In brief, mucus-bound *O*-glycans were released using Carlson’s reductive β-elimination procedure (heating at 45°C in 100 μL 50 mM NaOH containing 1 M NaBH_4_ for 16 h). Samples were briefly cooled on ice and neutralized by adding 10 μL aliquots of 2 M acetic acid, vortex-mixing after each addition, until evidence of bubbling stopped. Samples were diluted with ultrapure, 18.3 MΩcm H_2_O and partially *in vacuo* on a Savant SPD121P SpeedVac concentrator connected to a Savant RVT5105 refrigerated vapor trap (ThermoFisher Scientific) before they were snap frozen and lyophilized to dryness. *O*-glycans were recovered from the dried material by solid phase extraction (SPE) using 250 mg Supleco ENVI-Carb graphitic carbon cartridges (SigmaMillipore). Prior to use, SPE cartridges were conditioned with 3 mL aqueous 80% acetonitrile (ACN) with 0.1% trifluoroacetic acid (TFA) followed by 6 mL ultrapure H_2_O; positive pressure at the top of the tube was used for all SPE procedures. Crude glycan samples were re-dissolved in 500 μL ultrapure H_2_O and loaded onto the cartridges; an additional 200 μL of ultrapure H_2_O was used to rinse the sample tube and similarly applied to the SPE cartridge. Cartridges were washed with ultrapure H_2_O (3 mL), and reduced O-glycans were eluted using 50% ACN with 0.1% TFA (4 × 550 μL). Eluates were concentrated *in vacuo* to approximately 50 μL, transferred to 200 μL polypropylene HPLC vial inserts, and lyophilized. Samples were re-dissolved in 30 μL ultrapure H_2_O prior to analysis.

### Glycan high-performance liquid chromatography – mass spectrometry (HPLC-MS)

HPLC was conducted on an Agilent 1290 Infinity system (Agilent Technologies) with a 1290 Infinity binary pump, a 1290 Infinity autosampler, and a 1290 Infinity column compartment as previously desctibed.^127^ Analytes were separated on a Hypercarb 100 mm × 2.1 mm column (3 μm particle size; ThermoScientific) with the column temperature maintained at 60°C. Samples were analyzed using an injection volume of 5 μL and a flow rate of 0.450 mL/min. Mobile phases A and B were H_2_O and ACN, respectively, albeit data were collected using two different mobile phase additives previously used for glycomic analyses: 0.1% formic acid^68^ or 0.1% ammonium formate, pH 9.0.^70,71^ Gradient elutions (for both ammonium formate and formic acid-containing mobile phases) were programmed as follows: 0–15 min, 0–15% B; 15–22.5 min, 15–25% B; 22.5–25 min, 25–40% B; 25–25.2 min, 40–98% B. The column was then washed with 98% B for 2.6 min and re-equilibrated with mobile phase A for 3 min prior to the next injection. MS was conducted using an Agilent 6530 QToF-MS with an Agilent Jet Stream source operating in negative electrospray ionization mode. Source parameters were as follows: drying gas (N_2_) temperature of 300°C with a flow rate of 10 L/min; sheath gas (N_2_) temperature 400°C with flow rate of 12 L/min; nebulizer pressure 45 psig; capillary voltage 4750 V; nozzle voltage 1000 V; and fragmentor voltage 175 V. Reference ions containing 10 μM purine (*m*/*z* 119.0360 for [M-H]^−^) and 2.0 μM HP-0921 (*m*/*z* 966.0007 and 1033.9881 for [M-H]^−^ and [M+HCOO]^−^, respectively) in 95:5 ACN:H_2_O were added post-column using an Agilent 1260 Infinity II isocratic pump set to 8 μL/min. The QToF was tuned and calibrated in the 2 GHz extended dynamic range mode for the 100–3200 *m*/*z* range immediately prior to sample analysis. Full-scan spectra were collected at a rate of 2 Hz with a mass range of 100–3200 *m*/*z* and all data were saved in profile format.

HPLC-MS data acquisition and analysis were performed using MassHunter Workstation software (Agilent Technologies): Data Acquisition Workstation (v B.06.01, SP1) and Qualitative Analysis (v B.07.00, SP2). Specifically, MassHunter’s Find-by-Formula (FbF) algorithm was used to detect formulas consistent with putative glycans within a ±10 ppm mass accuracy limit and, when applicable, a ±0.175 min retention time window when the same peak was present in multiple samples. A FbF database was constructed with formulas corresponding to combinations of one or more of the following *m*/*z*-resolvable carbohydrate residues: *N*-acetylhexosamine (HexNAc), hexose (Hex), deoxyhexose (presumed to be fucose; Fuc), or *N*-acetylneuraminic acid (Neu5Ac), plus or minus sulfonate (SO_3_) moieties. Putative *O*-glycans were all assigned unique seven-digit glyco-names wherein each digit corresponded of HexNAc, Hex, Fuc, Neu5Ac, or sulfate moieties (in that order), while the _1, _2, (*etc*.) suffix denotes different HPLC-resolved isobars. At a minimum, all combinations contained one reduced HexNAc. Peak areas and retention times produced by FbF were processed further in Microsoft Excel. To account for differences in sample masses, peak areas for all detected glycans were normalized to the total glycan signals for each sample. All samples were analyzed in triplicate and mean relative HPLC-MS peak areas are reported.

### Glycan CE-LIF

CE-LIF was done for glycomics analysis as previously described.^127^ Novotny’s non-reductive, ammonia-catalyzed β-elimination procedure was used to cleave *O*-glycans from mucins in their reducing forms.^128,129^ In brief, mucin samples, freeze-dried into 1.5 mL screw-capped centrifuge tubes, were suspended in 750 μL 28% ammonium hydroxide containing 100 mg/mL ammonium carbonate; brief (<5 min) heating to 60°C followed by sonication in a water bath sonicator (VWR) was required to effectively dissolve all ammonium carbonate and thoroughly dispersed the mucin samples before they were heated for an additional 40 h. Samples were periodically vortexed during this period, after which they were cooled and concentrated on a SpeedVac centrifugal concentrator. The glycan- and ammonium salt-containing residue was resuspended in 500 μL H_2_O, briefly centrifuged (10,000 × g, 5 min, 20°C) to remove insoluble material and subjected to ENVI-Carb SPE exactly as described for the *O*-glycan alditols above. Note that the acidic SPE washes effectively convert glycans into their hemiacetal (reducing) forms that could be fluorogenically derivatized using 8-aminopyrene-1,3,6-trisulfonate (APTS) exactly as previously described.^123^ APTS-labeled glycans were electrophoretically resolved by CE-LIF on a ProteomeLab PA800 (Beckman-Coulter) using 50 cm × 50 μm internal diameter (44 cm to detector) fused silica capillary (Molex; Digi-Key). The background electrolyte was 25 mM ammonium acetate, pH 4.75, containing polyethylene oxide as an electroosmotic flow modifier (N-CHO buffer; SCIEX). Electrophoresis was carried out under reversed polarity (−30 kV) with samples being hydrodynamically injected (0.5 psi) at the cathode. Complete CE-LIF details have been previously described elsewhere.^130^

### ROS analysis

To inhibit ROS *in vitro*, cells were incubated with 10 μM of diphenyleneiodonium (80051–406, Calbiochem) overnight. ROS was measured with and without DPI with the DCFDA kit for ROS quantification as per the manufacturer’s instructions (ab113851, Abcam). The protocol was repeated, and cells were fixed and processed for confocal microscopy imaging as described above.

### Preparation of mucin granules

Mucin granules were prepared from confluent WT and *Mut* LS174T cells as previously described.^43^ In brief, cells were washed, harvested, and centrifuged. The resulting cell pellet was resuspended in MES homogenization buffer and homogenized with a Dounce homogenizer. The homogenate was then centrifuged twice to prepare a cleared supernatant, which was then centrifuged at 2,100 x *g* for 15 min resulting in crude granules. The mucin granules were further purified by resuspending in 2 mL HEPES buffer carefully placed on top of a sucrose gradient ranging from 2 M sucrose to 0.5 M sucrose and ultracentrifuged at 100,000 x *g* overnight. Following centrifugation, 1 mL fractions were isolated, washed three times in PBS, and used for further analysis.

### Soft agar colony formation assay

Tumor growth was analyzed by a soft colony formation assay as previously described.^79,131^ Agarose (0.6%) dissolved in serum-free EMEM was plated in 6-well plates and allowed to solidify overnight. The following day, 1 × 10^4^ cells were resuspended into a final concentration of 0.3% agarose dissolved in complete EMEM and layered on top of the 0.6% agarose base. After the layer solidified, 300 μL of complete media were put onto the plate and replaced every 3 d to prevent drying. Weekly, agar plates were imaged on the CellCyte X imager (Cytena), and tumor growth was analyzed using the built-in image analysis for spheroid growth. After 4 weeks, the plates were stained with 0.005% crystal violet in 10% alcohol for 1 h and destained in water overnight at 4°. Once destained, the plates were imaged with the bright light filter on a Bio-Rad ChemiDoc Imager. Tumor size and number were calculated in Fiji (ImageJ).

### Bead penetration assay

For the bead penetration assay, 0.2 and 1 μm fluorescent beads (FluoSpheres Sulfate Microspheres, Invitrogen) were inoculated on 24-well plates of 5-day-old WT and *Mut* cells at a concentration of 12 μL of beads/300 μL media for each well. After the appropriate time point, the media containing the beads were aspirated, the wells were washed thrice with cold PBS, and the plates were read on a fluorescence reader (SpectraMax i3x, Molecular Devices). Three hundred microliter of the bead mix in PBS was read as a measurement of total beads inoculated, and a ratio of remaining beads to total beads was calculated to determine what percentage of beads remained after washing. For confocal imaging, the same protocol was followed for 60 min before cells were fixed and processed according to the confocal protocol listed above.

### Bacterial growth

Wildtype *Salmonella enterica* strain SL1344 and a less invasive mutant of SL1344 (ΔinvA) (strain invA:kan SB103; SPI-1; a kind gift from Dr Bruce Vallance, Vancouver, Canada) were used. Small cultures of bacteria were established the day before adhesion or invasion assays (1 colony in 2.5 mL Luria-Bertani (LB) supplemented with 50 μg/mL streptomycin as both strains are streptomycin resistant). Overnight culture (800 μL) was sub-cultured into fresh LB (22 mL) and incubated for ~1.5 h (37°C, ~225 rpm). Optical density at a wavelength of 600 nm (OD600) was used to determine and adjust the concentration of bacteria to 5.0 × 10^8^ colony forming units (CFU)/mL in EMEM.

### Adhesion and invasion assays

Adhesion and invasion assay were based off gentamicin protection assays as previously reported.^132^ In the invasion assay, cell monolayers on 12-well plates were incubated with 0.5 mL/well of EMEM containing SalWT or ΔinvA bacteria (5.0 × 10^8^ CFU/mL), or 0.5 mL/well of control EMEM for 1 h at physiological conditions (37°C; 5% CO_2_) to promote invasiveness.^132^ After incubation, the culture supernatants were collected and stored on ice, and non-adherent bacteria were washed off with 3 PBS washes. Fifteen minutes after the removal of the supernatants, cells were incubated with gentamicin for 40 min (100 μg/mL in EMEM; 37°C; 5% CO_2_) to kill extracellular bacteria. After three more washes, cells were lysed in Triton buffer (300 μL; 0.5% Triton X-100; 0.1% sodium dodecyl sulfate [SDS]) and sonicated for 10 min to release the intracellular contents and invaded bacteria into the lysates, which were then stored on ice. The adhesion assay was conducted similarly, except the 1-h incubation was at room temperature instead of 37°C, and gentamicin was not applied; instead, after nonadherent bacteria were washed off, cells were lysed (300 μL; 0.1% Triton X-100; 0.1% SDS) and sonicated to release adhered bacteria and intracellular contents. To enumerate bacterial adhesion and invasion by cell type, lysates from both the invasion and adhesion assays were serially diluted in PBS and plated for CFU on LB-agar plates supplemented with streptomycin (50 mg/mL), as described elsewhere.^133^ Alternatively, cell supernatant was collected and processed for Western blotting, and the lysates were lysed with either RNA or protein lysis buffer and prepared for analysis as described above. For confocal imaging, the invasion protocol was followed for 60 min before cells were fixed and processed according to the confocal protocol listed above.

### LDH cytotoxicity assay

To determine cell death in response to *S. enterica*, a LDH cytotoxicity assay was performed on the supernatants following the invasion assay using the CytoTox-ONE Homogeneous Membrane Integrity Assay (Promega). The assay was performed as per the manufacturer’s instructions. Briefly, 40 μL of cleared supernatant from cells with or without bacteria was put into individual wells of a 96-well plate and mixed with 40 μL of assay buffer. The plate was left for 10 min at room temperature before reading on a fluorescence reader at 560 nm/590 nm (SpectraMax i3x, Molecular Devices). LDH release was compared to total cellular lysates and using the formula (sample LDH release – background reading)/(total sample lysates LDH – background reading) ×100 to get a percentage of LDH release.

### Wound assay

For the *in vitro* wound healing assay, WT or *Mut* cells were grown in culture inserts (ibidi Culture-Insert 2 well, Thermo Fisher Scientific). Cells were grown for 2 d with the insert until confluency, at which point the insert was removed, creating a clean, cell-free, and well-defined 500 μm cell-free gap. The cells were then allowed to heal for up to 7 d. The distance between the cells was measured daily, and cells were fixed temporarily for confocal analysis or cell lysates processed for RT-PCR.

## Statistical analysis

All experiments were repeated at least three independent times. Statistical analyses were done using GraphPad Prism 10 (Graph-Pad Software, San Diego, CA). Two-way ANOVA was used to determine statistical significance between more than two groups. Comparisons between two groups was done with the Student’s t-test. Slopes of mRNA and protein half-lives were calculated by linear regression and compared by analysis of covariance (ANCOVA). Significance was assumed at *p* < 0.05. Error bars depict mean ± standard error mean (SEM).

## Supplementary Material

Supplementary Figure Legends.docx

Supplementary Table Legends.docx

## Data Availability

The supplementary data that support the findings of this study are openly available at https://figshare.com/articles/dataset/Supplementary_Data_Set/28485530
