# Supplementary material for: Using MUC2 mucin producing tumorigenic human goblet-like cells to uncover functional properties of the mucus barrier
Source: Gut Microbes. 2025 Aug 8;17(1):2542385. doi: 10.1080/19490976.2025.2542385 (PMC12931731; doi:10.1080/19490976.2025.2542385)
Supplement: Supplementary Figure Legends.docx [file KGMI_A_2542385_SM6920.docx]

**Supplementary Figure 1.** *Mut* cell lysates and granules display an altered glycomics profile than WT when analyzed with an acidic mobile phase. **(A)** Pie chart of acidic (sialylated or sulfated) vs. neutral glycans for WT and *Mut* cells lysates and granules. **(B)** Pie chart of relative abundance of glycan classes for WT and *Mut* cell lysates and granules. **(C)** Heatmap of log2-transformed relative abundances of individual sialylated glycans found in WT and *Mut* cell lysates. Glycans with the same composition but unique isobars are indicated with an underscore and number. Fucosylated glycans are marked in red. Grey indicates the glycan was not present. **(D)** Heatmap of log2-transformed relative abundances of individual non-sialylated glycans found in WT and *Mut* cell lysates. Glycans with the same composition but unique isobars are indicated with an underscore and number. Fucosylated glycans are marked in red. Grey indicates the glycan was not present. **(E)** Venn diagram of overlapping and unique glycan structures identified in WT and *Mut* cells. Glycan numbers are indicated, and the percentage of total analyzed is indicated in parentheses. **(F)** Heatmap of log2-transformed relative abundances of individual sialylated glycans found in WT and *Mut* granules. Glycans with the same composition but unique isobars are indicated with an underscore and number. Fucosylated glycans are marked in red. Grey indicates the glycan was not present. **(G)** Heatmap of log2-transformed relative abundances of individual non-sialylated glycans found in WT and *Mut* cell lysates. Glycans with the same composition but unique isobars are indicated with an underscore and number. Fucosylated glycans are marked in red. Grey indicates the glycan was not present. **(H)** Venn diagram of overlapping and unique glycan structures identified in WT and *Mut* cells and granules. Glycan numbers are indicated, and the percentage of total analyzed is indicated in parentheses.

**Supplementary Figure 2.** *Mut* cells produce more growth factors and migrated and proliferated faster in response to wounding. **(A)** Culture media were collected on day 0 (removal of insert) and on days 2 and 4 after the wound insert removal. Samples were analyzed for growth factor production by Luminex multiplex assay (n=4 per day) *p<0.05, **p<0.01, ****p<0.0001. **(B)** Quantification of mean fluorescence intensity (MFI) of MUC2 and FCGBP compared to WT from **Fig. 12D** (n=12-16). *p<0.05, **p<0.01, ****p<0.000. **(C)** Confocal microscopy images of control WT and *Mut* cells during restitution showing paxillin (red), EdU (green) and DAPI (blue). Images are representative of 3 different experiments. Scale bar = 100 μm. **(D)** Quantification of mean fluorescence intensity (MFI) of paxillin and EdU incorporation compared to WT (n=12-16). ***p<0.001, ****p<0.0001 (n=15).
