# Supplementary material for: Using MUC2 mucin producing tumorigenic human goblet-like cells to uncover functional properties of the mucus barrier
Source: Gut Microbes. 2025 Aug 8;17(1):2542385. doi: 10.1080/19490976.2025.2542385 (PMC12931731; doi:10.1080/19490976.2025.2542385)
Supplement: Supplementary Table Legends.docx [file KGMI_A_2542385_SM6919.docx]

**Supplementary Table 1.** High impact mutations in *Mut* cells (relative to wildtype) per snpEff predictions.

**Supplementary Table 2.** Lines of evidence for gene function modifiers in MUC2-related genes across three modalities: genomics, proteomics, and targeted PCR.

**Supplementary Table 3.** Shotgun proteomics proteins from WT and *Mut* LS174T goblet-like cell lysates.

**Supplementary Table 4.** Glycomics analysis from whole cell lysates and purified mucin granules of WT and *Mut* LS174T goblet-like cells.
